# Supplementary material for: Precursor engineering of hydrotalcite-derived redox sorbents for reversible and stable thermochemical oxygen storage
Source: Nat Commun. 2022 Aug 30;13:5109. doi: 10.1038/s41467-022-32593-6 (PMC9427752; doi:10.1038/s41467-022-32593-6)
Supplement: Supplementary file 1 — Supplementary information [file 41467_2022_32593_MOESM1_ESM.pdf]

Supplementary Information for

Precursor engineering of hydrotalcite-derived redox sorbents for reversible and stable thermochemical oxygen storage

Michael High[1,#], Clemens F. Patzschke[1,#], Liya Zheng[1,#], Dewang Zeng[1,2], Oriol Gavalda-Diaz[3,4], Nan Ding[1], Ka Ho Horace Chien[1], Zili Zhang[1], George E. Wilson[3], Andrey V. Berenov[3], Stephen J. Skinner[3], Kyra L. Sedransk Campbell[5], Rui Xiao[2]*, Paul S. Fennell[1]*, Qilei Song[1]*

[1]Department of Chemical Engineering, Imperial College London, Exhibition Road, London, SW7 2AZ, UK
[2]Key Laboratory of Energy Thermal Conversion and Control (Ministry of Education), School of Energy and Environment, Southeast University, Nanjing 210096, PR China
[3]Department of Materials, Imperial College London, Exhibition Road, London, SW7 2AZ, UK
[4]Composites Research Group, University of Nottingham, Jubilee Campus, Nottingham, NG7 2GX, UK
[5]Department of Chemical and Biological Engineering, The University of Sheffield, Western Bank, Sheffield S10 2TN, UK
[#]These authors contributed equally to this work.
*Corresponding authors: q.song@imperial.ac.uk; p.fennell@imperial.ac.uk; ruixiao@seu.edu.cn

Supplementary Table S1. Summary of representative oxygen carrier materials for chemical looping with oxygen uncoupling (CLOU) process.

Oxygen carrier material	Loading of metal oxides (wt%)	R_{O2} [a] (wt%)	Cycles	Operation Temperature (°C)	Preparation methods	Ref
Supported metal oxides						
$CuO/MgAl_2O_4$ derived from CuMgAl-LDH	~60	6.4	500	900	CuMgAl-LDH by coprecipitation	This work
$CuO/Al_2O_3/Na_xAl_yO_z$	55	5.0	120	800-1000	CuAl LDH with sodium contamination	1
CuO/Al_2O_3	60	6	n.a.	850-985	freeze granulation	2
	40	n.a.	5	900-925	freeze granulation	3
$CuO/\gamma\text{-}Al_2O_3$ [b]	33	3.3	n.a.	900-1000	impregnation	4
$CuO/\alpha\text{-}Al_2O_3$ [b]	15	1.5	n.a.	900-1000	impregnation	4
CuO/ZrO_2	40	4	n.a.	900-1000	freeze granulation	5
	40	4	40	900-1000	mechanical mixing [c]	4
$CuO/MgAl_2O_4$	21	2.1	n.a.	900-1000	impregnation	5
	40	4	16	850-900	spray drying	6
	40	4	n.a.	900-925	freeze granulation	3
	60	6	40	900-1000	mechanical mixing	4
$CuO/CaAl_4O_7$	25-45	2.5-4.5	20	800-950	mechanical mixing	7
Natural materials						
Copper ore (CuO/SiO_2) [d]	87	8.7	-	700-1000	natural materials	8
	63	6.3	-	700-1000	natural materials	8
	6	0.6	20	700-1000	natural materials	8
Perovskite-type oxides						
$CaMn_{0.875}Ti_{0.125}O_3$	-	~1	45	800–1000	solid state reaction	9
$Ca_xLa_{1-x}Mn_{1-y}M_yO_{3-\delta}$ M=Mg, Ti, Fe or Cu	-	0.37-0.76	-	900–1000	solid state reaction	10
$SrFeO_3$		2.2		450-850	solid state reaction	11
$Sr_{1-x}Ca_xFeO_{3-\delta}$		2.2		550	metal-nitrate decomposition	12
Substituted $SrFeO_3$		0.99-1.47	1000	450-700	Doping on perovskite	13
Ca, Co doped $SrFeO_3$	-	1.2		400-500	Doping on perovskite	14
Other metal oxides (spinel and ferrites)						
Mg_2MnO_4	-	0.3	10	810-950	solid state reaction	15

Note: [a] gaseous O_2 releasing capacity, calculated from theoretical loading if not noted in the literature.
[b] formation of $CuAl_2O_4$ and consequently low O_2 releasing capacity.
[c] mechanical mixing followed by pelletizing by pressure, essentially mechanical mixing of commercial pure copper oxide powder (particle size ~10 μm) with other support (powder).
[d] particles with high loading of CuO agglomerated after one cycle.

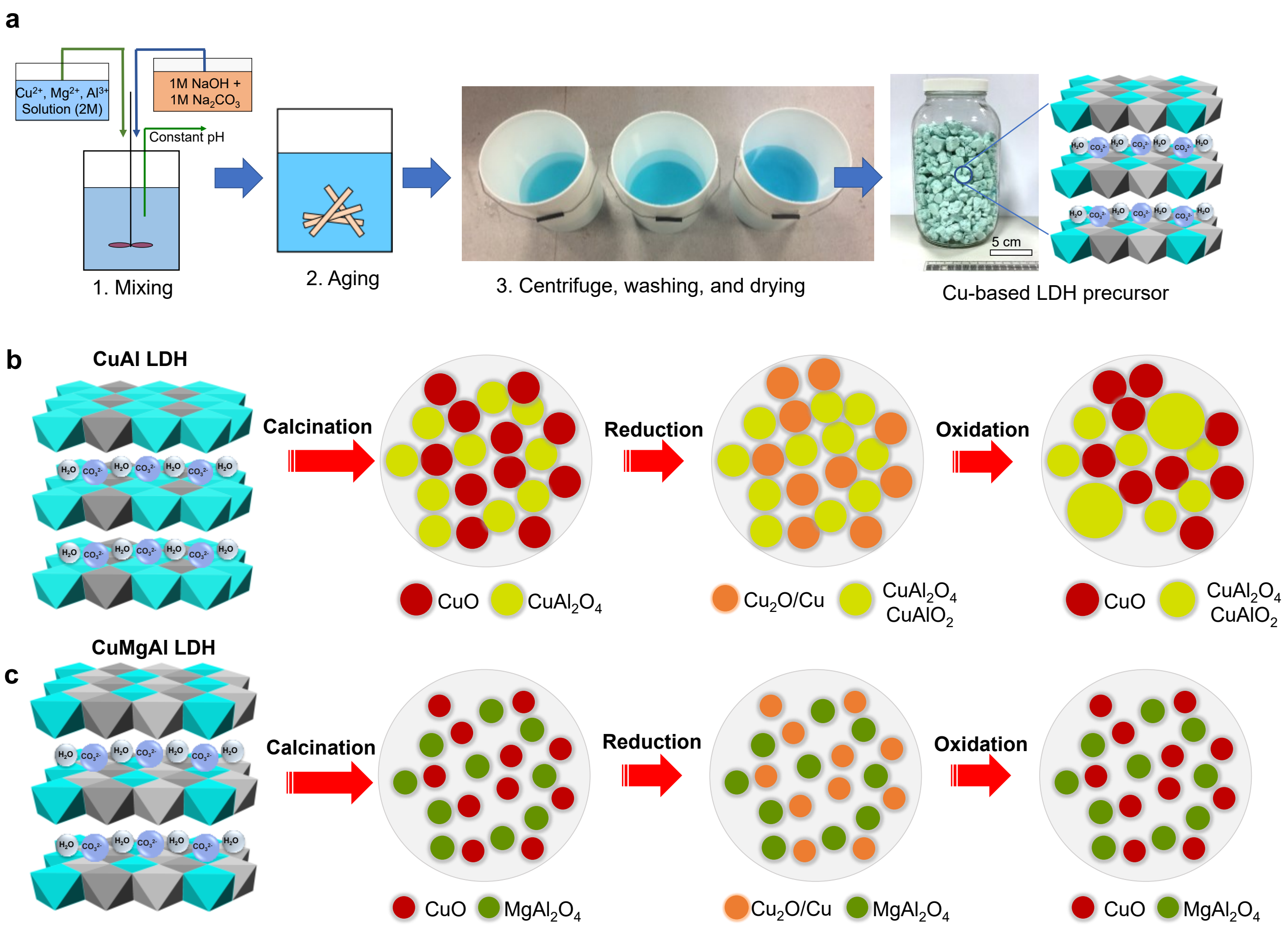

Supplementary Fig. S1. Synthesis of LDH precursors and derived redox sorbents. a, The LDH precursors were synthesised by co-precipitation at room temperature at a constant pH. The metal nitrate solution and the alkaline solution were co-added dropwise to a beaker under vigorous stirring by a magnetic stirrer (at about 300 rpm). The blue precipitate was aged for 3 h at room temperature followed by washing to remove excess sodium ions and vacuum filtration. The washing was stopped when the conductivity of the wash water measured below 50 $\mu S \cdot cm^{-1}$. After vacuum filtration, the collected blue solid product was dried in a ventilated oven at 60 °C for at least 12 h. A portion of dried precursor was retained for material characterisation. The rest of the dried precursor was calcined in a tube furnace at 950 °C for 3 h using a heating rate of 15 $°C \cdot min^{-1}$ under an air flow of 1 L min^{-1}. After cooling to room temperature, the solids were crushed using a pestle and mortar and sieved to the desired size fractions for testing and characterisation. The synthesis was scaled up to kilogram scale. (**b**) Calcination of CuAl-LDH and (**c**) CuMgAl-LDH precursor to mixed metal oxides. The binary CuAl-LDH precursor forms a mixture of CuO and $CuAl_2O_4$ after calcination at high temperature. For tertiary CuMgAl-LDH, the addition of Mg to the LDH structure inhibited the formation of $CuAl_2O_4$ by the preferential formation of $MgAl_2O_4$ which acts as a stable support for CuO by inhibiting sintering and agglomeration during redox cycling.

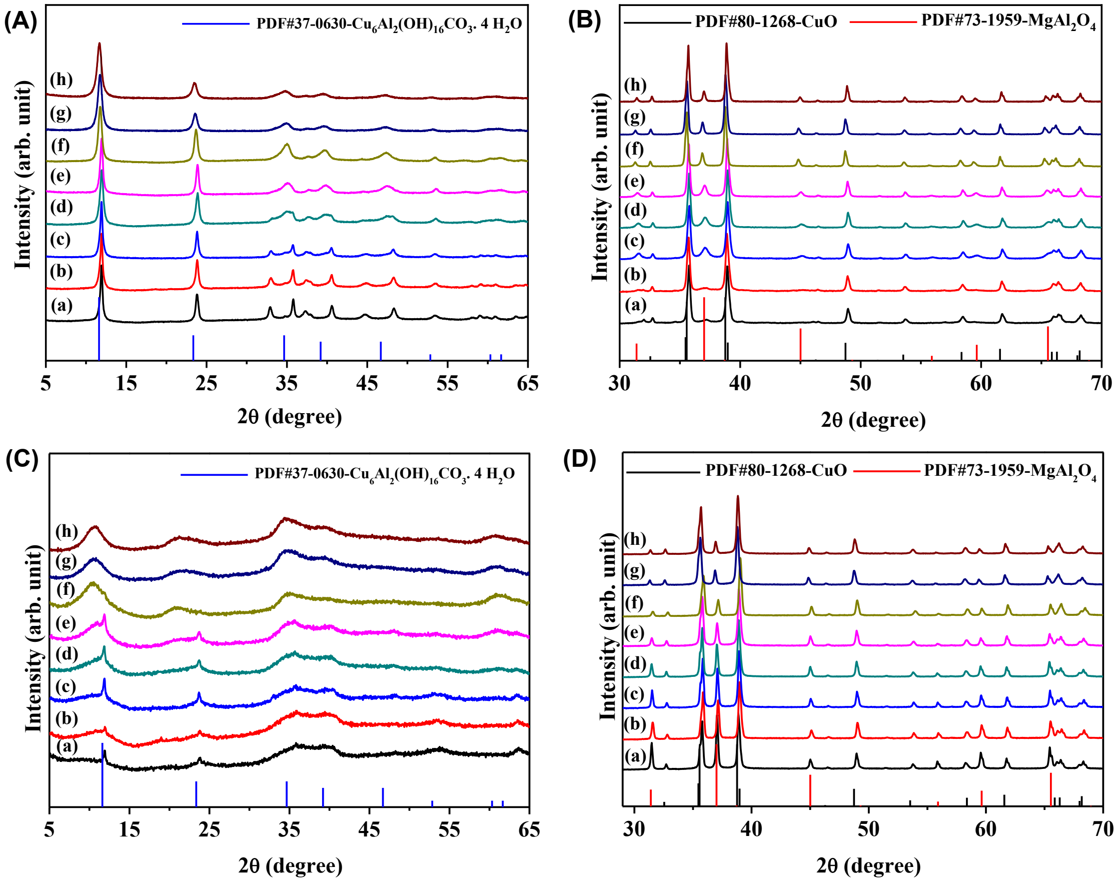

Supplementary Fig. S2. XRD patterns of LDH precursor and calcined mixed metal oxides. (A-D) XRD patterns of **(A, C)** the precursors (LDH) and **(B, D)** calcined mixed metal oxides co-precipitated using an aqueous solution containing 1 M **(A, B)** Na_2CO_3 + NaOH in an equimolar ratio and **(C, D)** 2M NaOH. The samples shown in **(A-D)** have the following nominal molar compositions: **(a)** Cu:Mg:Al = 3 : 0 : 2, **(b)** Cu:Mg:Al = 3 : 0.1 : 2, **(c)** Cu:Mg:Al = 3 : 0.2 : 2, **(d)** Cu:Mg:Al = 3 : 0.5 : 2, **(e)** Cu:Mg:Al = 3 : 0.8 : 2, **(f)** Cu:Mg:Al = 3 : 1 : 2. **(g)** Cu:Mg:Al = 4: 1 : 2. **(h)** Cu:Mg:Al = 5 : 1 : 2. The LDH precursors were calcined into mixed metal oxides at 950°C for 3 h.

Supplementary Table S2.

Interplanar spacing and crystallite size calculated using Scherrer's equation (L= K λ / (B cos (θ)) with K = 0.9 and λ = 0.15406 nm. The peak broadening of the instrument was determined experimentally as 0.102 FWHM (°2θ) using the (111) peak of a Si standard.

molar Cu:Mg:Al ratio	sample shown in Fig. 2	LDH		MMO	
		d_{003} [nm]	L_{LDH} [nm]	L_{CuO} [nm]	$L_{MgAl2O4}$ [nm]
3:0:2	(1)	0.746 ± 0.001	27.3 ± 5	37 ± 6	-
3:0.1:2	(2)	0.745 ± 0.001	24.1 ± 2	54 ± 13	-
3:0.2:2	(3)	0.746 ± 0.001	27.3 ± 5	39 ± 13	18.6 ± 1.7
3:0.5:2	(4)	0.746 ± 0.001	21.9 ± 4	44 ± 9	22.1 ± 2.4
3:0.8:2	(5)	0.745 ± 0.002	21.9 ± 4	44 ± 9	28.2 ± 3.7
3:1:2	(6)	0.737 ± 0.001	21.9 ± 4	39 ± 10	43.9 ± 8.8

The *d*-spacing, *d*, of the LDH was calculated using the XRD data shown in Supplementary **Fig. 2a** and the Bragg's law ($\lambda = 2d$ (sin θ). In addition, the crystallite size, L, was calculated for the LDH and MMO using Scherrer's equation (L = K λ / (B cos (θ)) and the data shown in Supplementary **Fig. 2a** and **Fig. 2b**, respectively.

Supplementary Table S3.

The composition of LDH precursors and derived mixed metal oxides, prepared with 1 M NaOH + 1 M Na_2CO_3 at a pH of 9.5 calculated from XRF and ICP results. Most of the variations are within a range of 5% measurement error.

Sample	Theoretical (wt%)			LDH								MMO			
				ICP results				XRF				XRF			
**Cu: Mg: Al**	**CuO**	**MgO**	**Al_2O_3**	**CuO**	**MgO**	**Al_2O_3**	**Na_2O**	**CuO**	**MgO**	**Al_2O_3**	**Na_2O**	**CuO**	**MgO**	**Al_2O_3**	**Na_2O**
**3: 0: 2**	70.06	0.00	29.94	69.53	0.00	27.55	2.91	64.89	0.17	32.75	2.18	61.84	0.18	34.69	3.29
**3: 0.1: 2**	69.24	1.17	29.59	68.96	0.78	27.92	2.33	62.83	1.03	34.40	1.74	62.68	1.04	32.36	3.92
**3: 0.2: 2**	68.44	2.31	29.24	70.66	1.15	25.86	2.34	64.09	1.95	31.73	2.23	69.42	1.33	27.94	1.31
**3: 0.5: 2**	66.15	5.59	28.26	70.44	3.20	25.53	0.83	67.27	3.45	28.73	0.56	66.65	3.66	29.03	0.66
**3: 0.8: 2**	64.01	8.65	27.35	69.03	5.21	25.40	0.36	61.48	6.40	31.88	0.24	65.93	5.73	28.05	0.30
**3: 1.0: 2**	62.65	10.58	26.77	66.06	8.55	25.31	0.07	60.93	9.20	29.86	0.00	61.31	9.84	28.85	0.00
**4: 1.0: 2**	69.10	8.75	22.14	71.92	6.94	21.15	0.00	63.45	8.70	27.85	0.01	59.23	10.14	30.63	0.00
**5: 1.0: 2**	73.65	7.46	18.88	77.75	4.39	17.86	0.00	72.53	5.84	21.62	0.01	73.67	6.16	20.17	0.00

Supplementary Table S4.

The composition of LDH precursors and derived mixed metal oxides, prepared with 2 M NaOH at a *pH* of 9.5 calculated from XRF and ICP results. Most of the variations are within a range of 5% measurement error.

Sample	theoretical (wt%)			LDH								MMO			
				ICP results				XRF				XRF			
Cu: Mg: Al	CuO	MgO	Al_2O_3	CuO	MgO	Al_2O_3	Na_2O	CuO	MgO	Al_2O_3	Na_2O	CuO	MgO	Al_2O_3	Na_2O
**3: 0: 2**	70.06	0.00	29.94	74.85	0.00	25.15	0.00	70.02	0.26	29.71	0.01	68.52	0.40	31.08	0.00
**3: 0.1: 2**	69.24	1.17	29.59	76.16	0.00	23.84	0.00	65.75	1.33	32.89	0.03	68.62	1.18	30.19	0.00
**3: 0.2: 2**	68.44	2.31	29.24	72.92	1.98	25.10	0.00	64.36	2.46	33.18	0.00	71.68	1.96	26.36	0.00
**3: 0.5: 2**	66.15	5.59	28.26	68.08	4.64	27.28	0.00	63.66	4.94	31.40	0.00	62.18	5.65	32.18	0.00
**3: 0.8: 2**	64.01	8.65	27.35	67.01	7.02	25.97	0.00	65.74	6.84	27.42	0.00	57.53	9.52	32.95	0.00
**3: 1.0: 2**	62.65	10.58	26.77	65.11	9.16	25.73	0.00	64.39	8.75	26.86	0.00	58.29	11.04	30.68	0.00
**4: 1.0: 2**	69.10	8.75	22.14	71.07	7.72	21.21	0.00	62.77	9.69	27.54	0.00	51.37	13.56	35.08	0.00
**5: 1.0: 2**	73.65	7.46	18.88	75.56	6.49	17.94	0.00	70.56	7.44	22.00	0.00	71.77	7.64	20.59	0.00

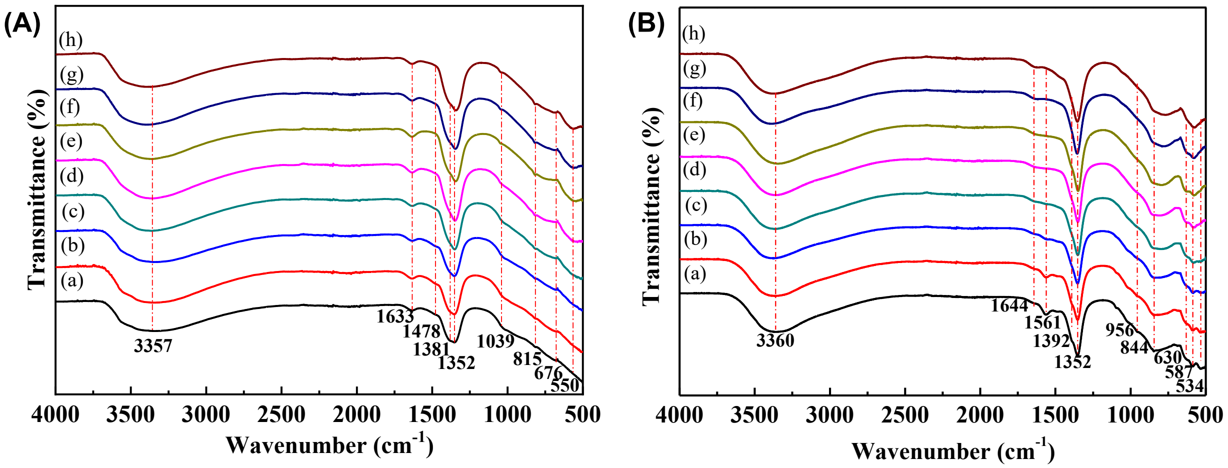

Supplementary Fig. S3. FTIR spectra of LDH precursors. **(A)** NaOH precipitation agent and **(B)** mixture of NaOH and Na_2CO_3, with varying molar compositions: **(a)** Cu:Mg:Al = 3 : 0 : 2, **(b)** Cu:Mg:Al = 3 : 0.1 : 2, **(c)** Cu:Mg:Al = 3 : 0.2 : 2, **(d)** Cu:Mg:Al = 3 : 0.5 : 2, **(e)** Cu:Mg:Al = 3 : 0.8 : 2, **(f)** Cu:Mg:Al = 3 : 1 : 2. All samples were prepared at a constant *pH* of 9.5. The peak at 1352 cm^{-1} associated with CO_3^{2-} is weaker for the precursors prepared with NaOH than for those prepared using NaOH + Na_2CO_3. Although Na_2CO_3 was not used as a precipitant for (A), CO_3^{2-} has an extremely high affinity to the LDH and is incorporated into the interlayer of the LDH due to CO_2 ingress during precipitation. Despite the incorporation of some CO_3^{2-} into the interlayer, the absence of the shoulder at 630 cm^{-1} demonstrates the quantity of CO_3^{2-} in the interlayer is lower than in the samples precipitated with NaOH + Na_2CO_3. Interlayer NO_3^- peaks at 1381 cm^{-1} and 1039 cm^{-1} are observed in (A) and the absence of these peaks in (B) demonstrate the interlayer anions of the LDH synthesised using NaOH are a mixture of NO_3^- and CO_3^{2-}, whereas the interlayers anions for the LDH prepared using NaOH + Na_2CO_3 are primarily CO_3^{2-}.

Fourier-transform infrared spectroscopy (FTIR) was performed using the KBr pellet technique. The broad absorption band at about 3357 cm^{-1} is assigned to the asymmetrical and symmetrical stretching vibrations modes of OH groups in the layers and the interlayer water molecules. A relatively weak absorption band at 1633cm^{-1} is assigned to the interlayer water deformation bending. The sharp band at 1352 cm^{-1} and the small shoulder at 630 cm^{-1} were assigned to the CO_3^{2-} anion. The peaks at 1381 cm^{-1} and 1039 cm^{-1} are NO_3^- anions in the interlayer. The series of peaks in the range of 800-400 cm^{-1} are related to the O– M and O–M–O vibrational modes (M = Al, Cu, Mg).

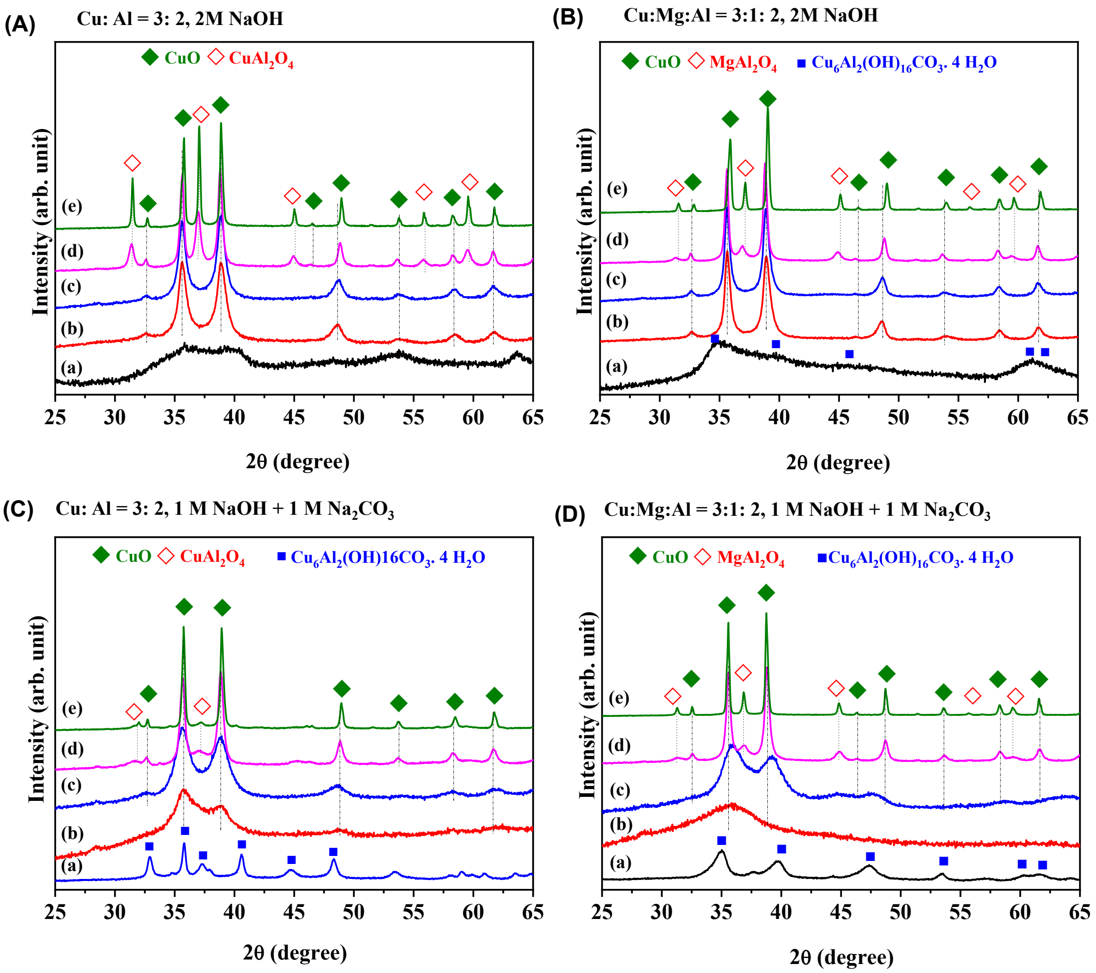

Supplementary Fig. S4. The phase composition as a function of the calcination temperature. (A) CuAl-LDH (Cu:Al=3:2), with precipitation agent of 2 M NaOH, (B) CuMgAl-LDH (Cu:Mg:Al = 3:1:2) with precipitation agent of 2 M NaOH, (C) CuAl-LDH (Cu:Al=3:2) with precipitation agent of 1M NaOH + 1M Na_2CO_3; (D) (Cu:Mg:Al = 3:1:2) with precipitation agent of 1M NaOH + 1M Na_2CO_3. CuO: JCPDS 80-1268. $CuAl_2O_4$: JCPDS 76-2295. $MgAl_2O_4$: JCPDS 73-1959. $Cu_6Al_2(OH)_{16}CO_3 \cdot 4H_2O$: JCPDS 37-0630. The treatment temperatures were the following: (a) room temperature, (b) 400 °C, 3 h (c) 600 °C, 3 h (d) 800 °C, 3 h (e) 950 °C, 3 h. LDH precursors were calcined for 3 hours at varying temperatures (as specified in the caption), cooled to room-temperature using an air flow of 1 l min^{-1} at SATP and analysed by XRD. The LDH precursors synthesised using NaOH (a-b) have a lower thermal stability than the precursors synthesised using NaOH and Na_2CO_3 (c-d), due to the formation of crystalline CuO at lower temperatures. For CuAl LDH prepared by precipitation reagent of NaOH and Na_2CO_3 (c), the formation of $CuAl_2O_4$ was limited by the presence of sodium-containing species.

Mechanism of inhibiting Na-containing species in LDH precursors by the presence of Mg-species

We believe that the formation of dawsonite, $NaAlCO_3(OH)_2$, plays an important role in formation of Na-containing species in the LDH precursors and derived mixed metal oxides. In the following we present and discuss the results of thermodynamic calculations as evidence of this relationship.

The thermodynamic calculations have been carried out using SPANA (formerly MEDUSA)[16], which is based on the procedures developed by Kakolowicz et al.[17] and Eriksson [18].

In the pH values range used during co-precipitation in this work (*i.e.* pH 9.5–11.0), the overall reaction for dawsonite formation is shown in eq.(1)[16], with its intermediates typically forming according to the reactions reactions eq.(2)–eq.(4).

$$Al^{3+} + Na^{+} + HCO_3^{-} + 2\ H_2O - 3H^{+} \leftrightarrow NaAl(CO_3)(OH)_2 \quad (1)$$
$$Al^{3+} + 3\ OH^{-} \leftrightarrow Al(OH)_3 \quad (2)$$
$$2\ NaOH + 2\ Al(OH)_3 \leftrightarrow 2\ NaAl(OH)_4 \quad (3)$$
$$NaAl(OH)_4 + CO_3^{-2} + 2\ H^{+} \leftrightarrow NaAl(CO_3)(OH)_2 + 2H_2O \quad (4)$$

Next, we have used SPANA, [16] to compare the predicted thermodynamic equilibrium in the presence and absence of Mg ions. A thorough screening of the available species in SPANA's standard database DATABASE (formerly HYDRA) yielded that some relevant compounds, such as dawsonite and bimetallic hydrotalcite-like compounds, are not included in this standard database. We have thus added these compounds using values for the critical stability constants (k_r) from the literature, as listed in Table S5.

Supplementary Table S5.

Values for the equilibrium constants (k_r) and structures for the compounds added to the dataset used for the thermodynamic calculations with SPANA.

Formula	Name	Log $k_{r,298K}°$	Reference (Database)
$Mg_2CO_3(OH)_2·3H_2O$	Artinite	19.66	[19, 20] (EQ3/EQ6)
$NaAlCO_3(OH)_2$	Dawsonite	4.35	[19, 20] (EQ3/EQ6)
$Mg_5(CO_3)_4(OH)_2·4H_2O$	Hydromagnesite	32.55	[20, 21] (MINTEQA2)
$Mg_4Al_2(OH)_{14}·3H_2O$	OH-Hydrotalcite	-56.02	[22-24]
$Mg_4Al_2\ (OH)_{14}·3H_2O$	OH-Hydrotalcite	-49.70	[22, 24-26]
$Mg_4Al_2(OH)_{12}(CO_3)·\ 2H_2O$	CO_3-hydrotalcite	-51.14	[22, 23, 27]

The results of the thermodynamic calculations using SPANA's standard database and additionally dawsonite and $Mg_4Al_2(OH)_{12}(CO_3)·2\ H_2O$ with data from Table S5, in the presence and absence of Mg are presented in Figure S5A and S5B, respectively.

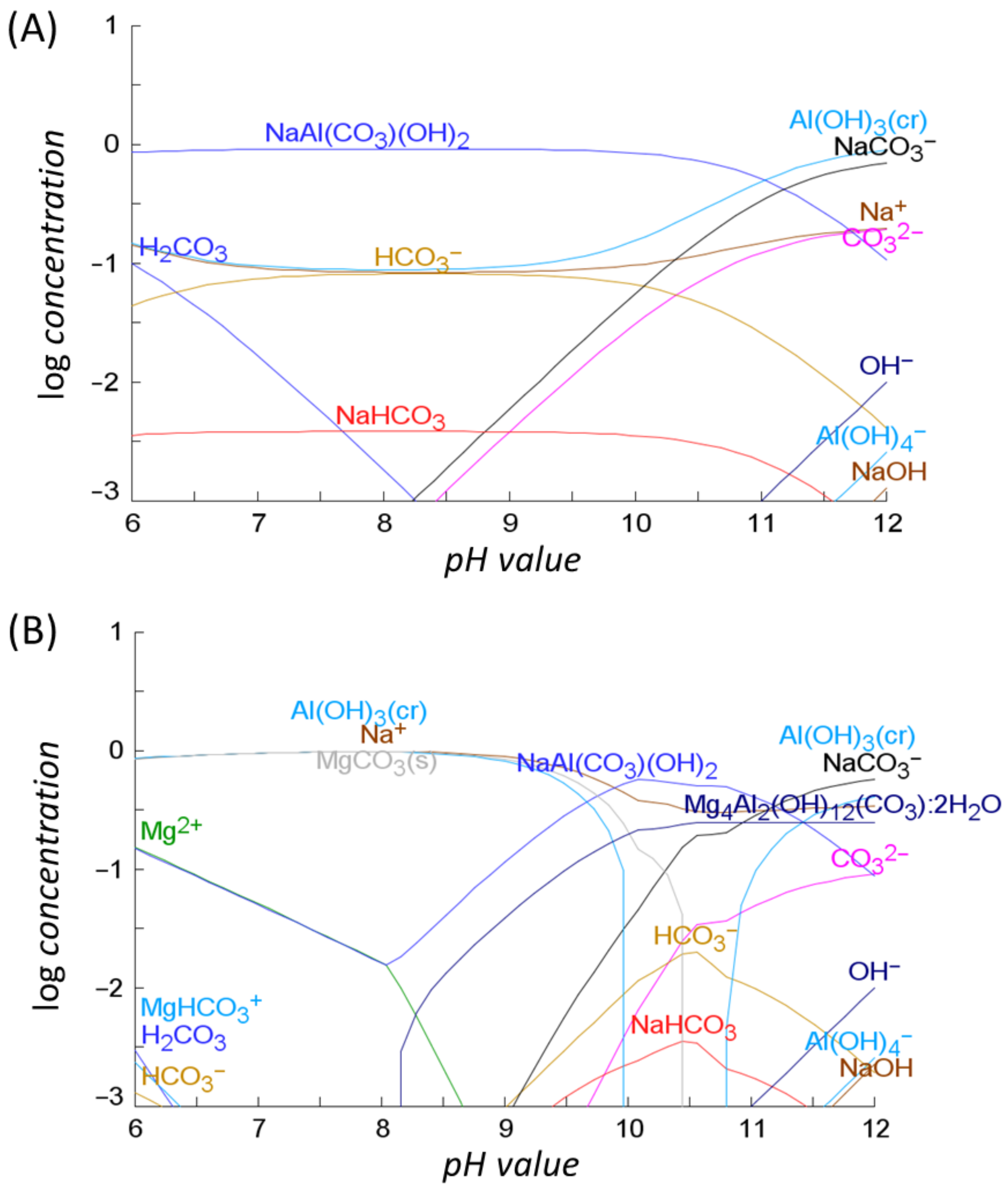

Supplementary Fig. S5. Plots of equilibrium compositions (A) in the absence and (B) presence of Mg ions as a function of the pH value. The plots were generated with SPANA (formerly MEDUSA) using data from DATABASE (formerly HYDRA) [16] as well as literature values for the compounds listed in Table S5. The following ion concentrations were used: (A): $[Na^+]=[Al^{3+}]=[CO_3^{2-}]=1$ M and (B): $[Na^+]=[Mg^{2+}]=[Al^{3+}]=[CO_3^{2-}]=1$ M.

Figure S5A shows formation of dawsonite over a wide pH value range. However, when the calculation was repeated with Mg ions being present additionally, as shown in Figure S5B, the predicted equilibrium molar fraction of dawsonite is significantly reduced at an intermediate pH (*e.g.* in the pH value range 7–9). Instead, $MgCO_3$ and $Al(OH)_3$ formation is favoured. These species are likely to function as precursors for MgAl-LDH formation (*c.f.* eqs.(7-11)). Above a pH value of 8.2, MgAl-LDH (e.g. Mg-Al

hydrotalcite) is predicted to form – though a high molar equilibrium fraction (log *concentration* > -1) is only predicted to occur at a pH value of > ca. 9.5. Although this equilibrium calculation does not directly show that formation of dawsonite is thermodynamically limited, it is likely that an interplay between a thermodynamic suppression of dawsonite formation (and formation of its precursors) and faster kinetics of the formation of intermediate species required for Mg-Al hydrotalcite formation and their combination to the hydrotalcite are leading to the inhibition of dawsonite formation. In experimental conditions, we used a mixture of NaOH and Na_2CO_3, which may be more favourable for the formation of MgAl-LDH structures instead of Na-Al dawsonite.

The hydrotalcite formation is possible *via* an ion exchange reaction with the $NaAl(OH)_4$, *e.g.* produced according to eq.(3), according to the reactions shown in eq.(7)–(8):

$$NaAl(OH)_4 + Mg^{2+} + 2\ H_2O = AlMg(OH)_5 + NaOH + 2\ H^+ \quad (7)$$

$$2\ NaAl(OH)_4 + 4\ Mg^{2+} + 11\ H_2O = Mg_4Al_2(OH)_{14}\ 3\ H_2O + 2\ NaOH + 8\ H^+ \quad (8)$$

Since hydrated sodium aluminate can also dissociate, a two-step reaction during which the dissociated aluminium hydroxide reacts with an Mg-ion is also a possible pathway:

$$2\ Na[Al(OH)_4] \rightarrow 2\ NaOH + 2\ Al(OH)_3 \quad (9)$$

$$Al(OH)_3 + Mg^{2+} + 2\ H_2O \rightarrow AlMg(OH)_5 + 2\ H^+ \quad (10)$$

$$2\ Al(OH)_3 + 4\ Mg^{2+} + 11\ H_2O \rightarrow Mg_4Al_2(OH)_{14} \cdot 3\ H_2O + 8\ H^+ \quad (11)$$

In these reactions, Mg-species are likely to effectively scavenge carbonate groups and Al from either dawsonite or from the mix of ions required for dawsonite formation. In a final stage, the positively charged Al-Mg hydroxide forms two-dimensional layers while intercalating carbonate groups according to the reaction such as eq.(12) as predicted by SPANA:

$$Mg_4Al_2(OH)_{14} \cdot 3H_2O + CO_3^{2-} + 2\ H^+ \rightarrow Mg_4Al_2(OH)_{12}CO_3 \cdot 2H_2O + 3\ H_2O \quad (12)$$

Following the formation of the CO_3-Mg-Al layered double hydroxide, all Na remains soluble as Na^+ and $NaCO_3^-$ (*c.f.* Figure S5B). During the repeated DI-water washing of the precursor which is part of the synthesis procedure of this work, these soluble Na-species separate from the solids and are removed with the supernatant, thus explaining the inhibition of Na contamination in the tertiary CuMgAl-LDH system.

In a binary CuAl-LDH system, such as the one investigated by Song et al. 2013[1], the absence of Mg allowed dawsonite to form. The precipitation of dawsonite in the solid phase prevents removal of the dawsonite (or any Na from the precipitated dawsonite) during the subsequent DI washing procedure and the sodium remains as a contaminant in the calcined material.
The inhibiting effect of Mg on dawsonite formation has also been observed in natural subsurface dawsonite reservoirs. The latter appear to only exist in “Mg-poor environmental conditions”.[28] In lab experiments they [28] reproduced potential dawsonite formation conditions and have likewise observed an inverse relationship between [Mg] and the formation of dawsonite.

Thermodynamic calculations to elucidate the inhibiting effect of Mg on active phase–support interactions

To demonstrate the inhibiting effect of Mg on active phase–support interaction, we have carried out thermodynamic calculations with FactSage for the calcined system in the presence and absence of Mg.[29] This has been done for 600 °C and 1000 °C (and temperatures in-between) to cover a wide range of potential temperatures for CLC. Furthermore, the equilibrium composition has been plotted as a function of the oxygen concentration, since the mechanisms of active phase–support interaction for the Cu-Al-O system (which is prone to these interactions) does not become apparent in the fully oxidised state (Figure S6A and S6B with x in Cu + 2 Al + x O_2 = 2.0. Here, CuO and Al_2O_3 are thermodynamically stable. These two species are also stable in the fully reduced state (x = 1.5). However, at intermediate oxidation states or upon O_2 removal during thermal decomposition of CuO, a mixture of $CuAlO_2$, Al_2O_3 and Cu or CuO is thermodynamically stable. This allows for the unfavourable Cu-Al oxide formation, which has been observed in the literature (as discussed in the main article).

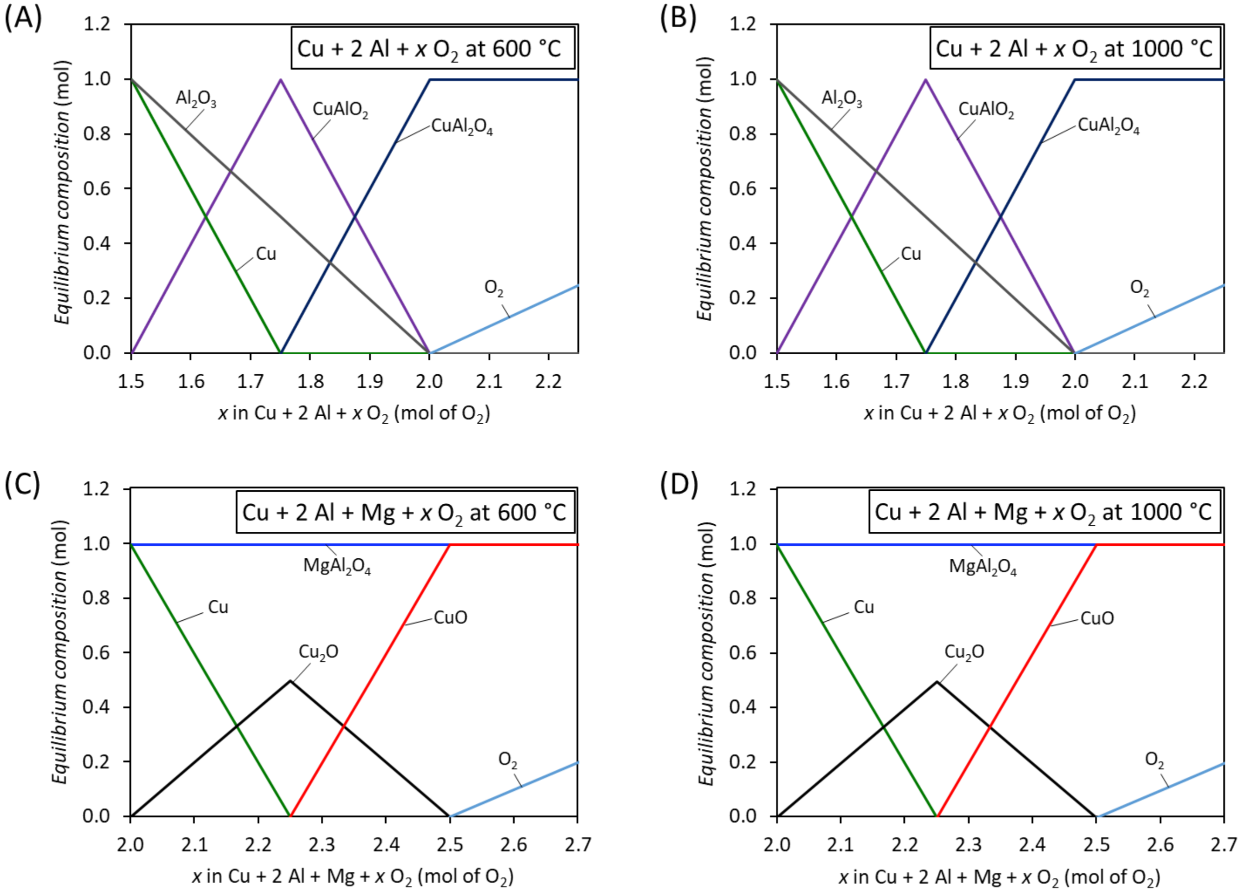

Supplementary Fig. S6. Plots of the thermodynamic equilibrium composition as a function of the oxygen concentration using data from FactSage and expanded with $CuAl_2O_4$ using Gibbs free energy data for the systems (A, B) Cu-Al-O and (C, D) Cu-Al-Mg-O, and the temperatures (A, C) 600 °C and (B, D) 1000 °C.

For the trimetallic-system Cu-Al-Mg-O (Fig. S6C and S6D), interactions between the active phase and the supporting phase are thermodynamically not favourable in the temperature range 600–1000 °C and oxygen concentrations in the range occurring between full reduction and oxidation, as shown in Figure S6C and S6D.[28]

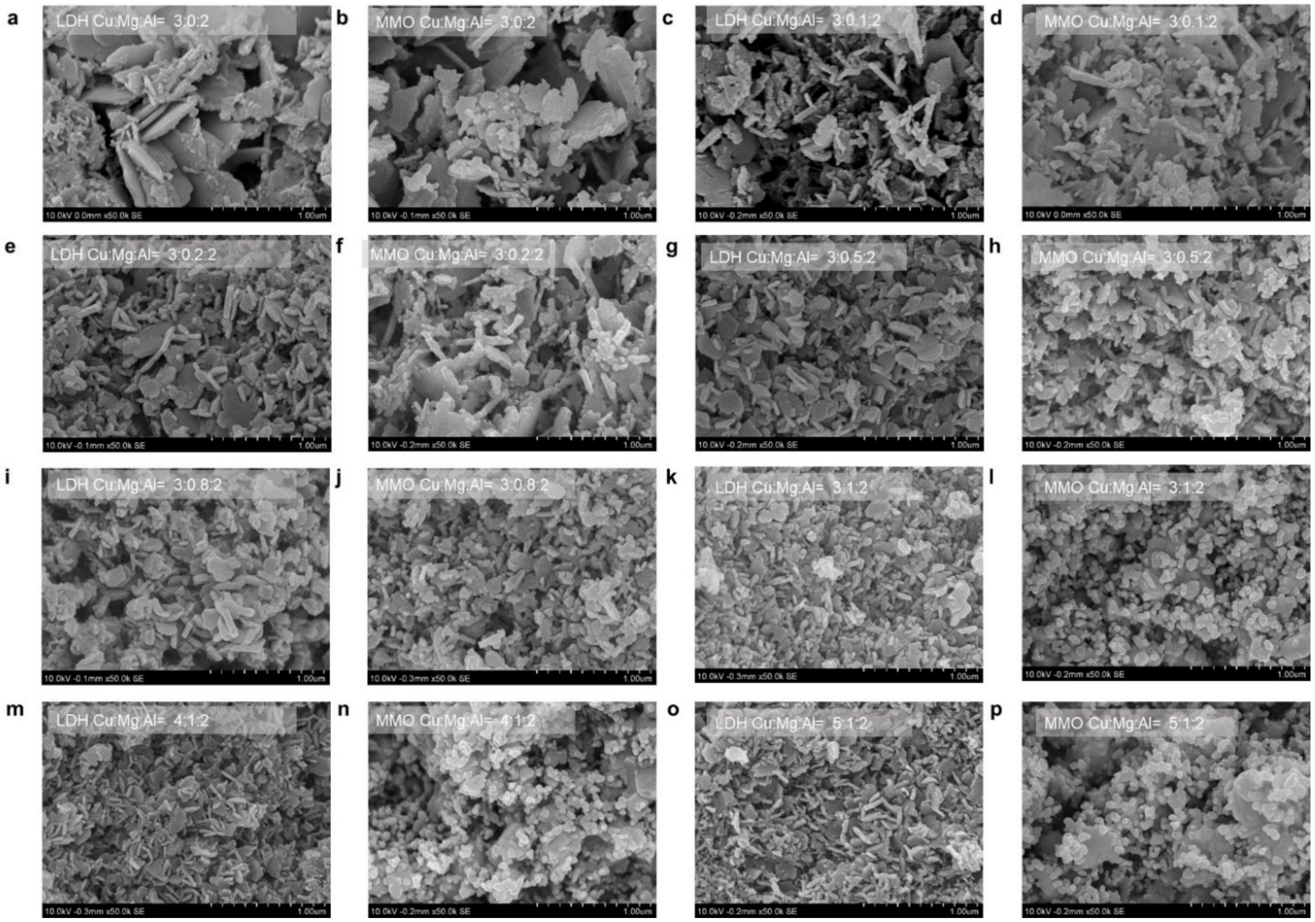

Supplementary Fig. S7. SEM images of LDH precursors synthesised at *pH* 9.5 using 1 M NaOH + 1 M Na_2CO_3 and calcined mixed metal oxides (MMO). (a-b) Cu:Mg:Al = 3 : 0 : 2, (c-d) Cu:Mg:Al = 3 : 0.1 : 2, (e-f) Cu:Mg:Al = 3 : 0.2 : 2, (g-h) Cu:Mg:Al = 3 : 0.5 : 2, (i-j) Cu:Mg:Al = 3 : 0.8 : 2, (k-l) Cu:Mg:Al = 3 : 1 : 2. (m-n) Cu:Mg:Al = 4 : 1 : 2. (o-p) Cu:Mg:Al = 5 : 1 : 2. With increasing Mg content, a decrease in the mean size of the LDH platelets and narrowing of the size distribution of LDH platelets is observed, i.e., the platelets become more uniform with increasing Mg content. Accordingly, the surface of the calcined samples becomes more uniform with increasing Mg content.

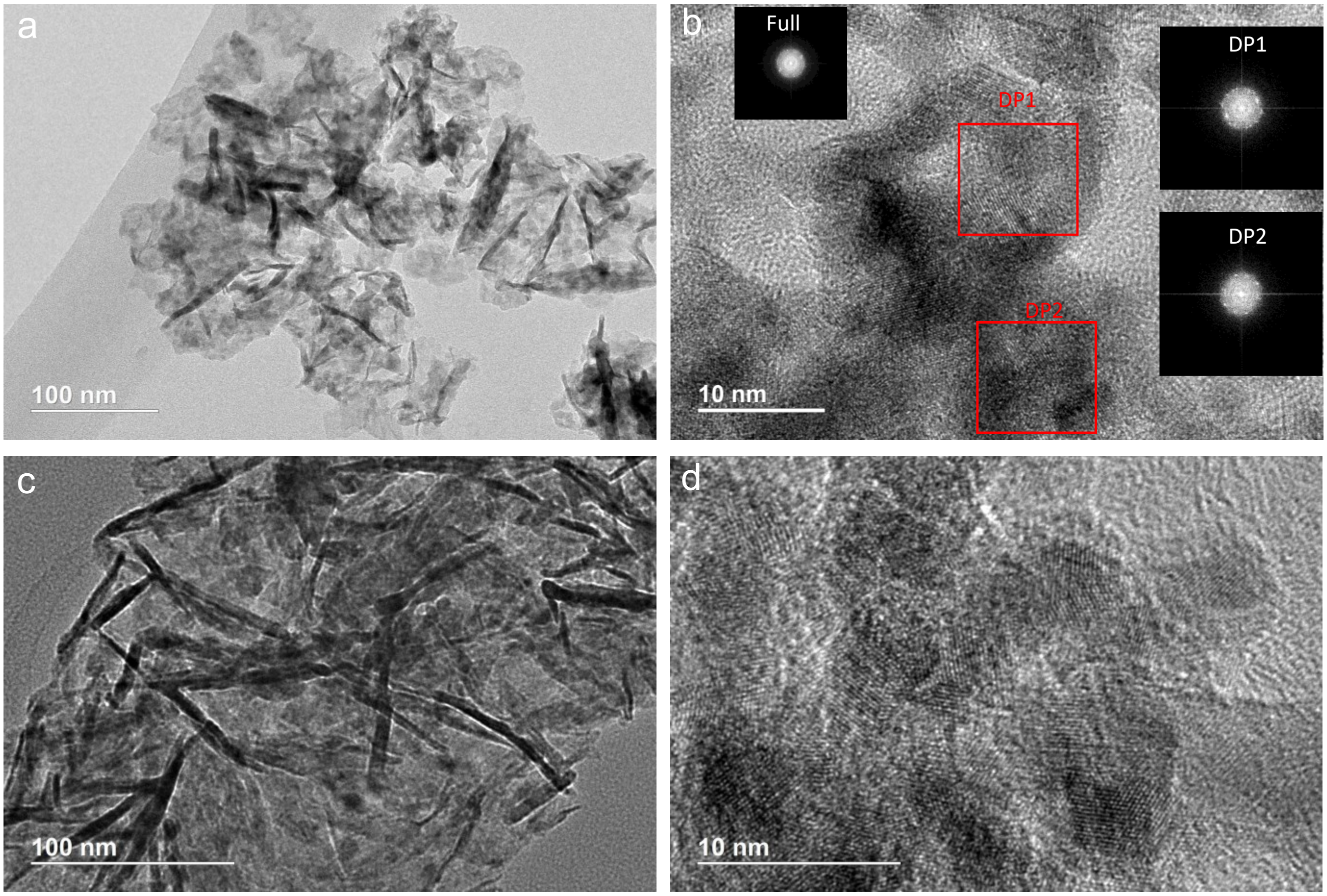

Supplementary Fig. S8. Characterization of CuMgAl-MMO. a, c, STEM image of CuMgAl-MMO. **b, d**, HR-TEM images of CuMgAl-MMO. The CuMgAl-MMO sample was prepared by heating up the CuMgAl-LDH (Cu:Mg:Al = 3:1:2) to 800°C and immediately cooled down to room temperature. The LDH precursor was precipitated with an aqueous solution containing 1M NaOH and 1M Na_2CO_3. The HR-TEM analyses confirmed the high dispersion of fine CuO particles (<10 nm) in the support owing to the high degree of dispersion in the CuMgAl-LDH precursor, with inhibited crystallization of CuO at high calcination temperature.

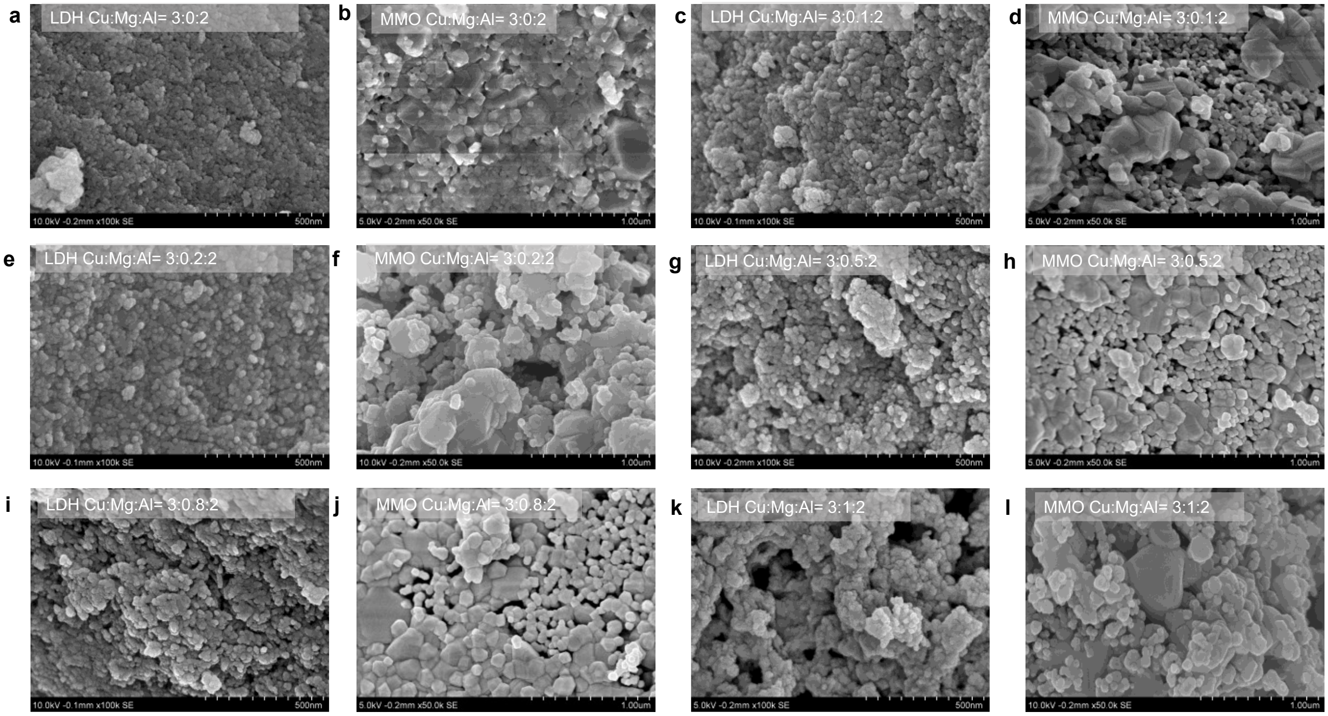

Supplementary Fig. S9. SEM images of LDH precursors and calcined mixed metal oxides prepared using 2M NaOH as precipitation agent. (a-b) Cu:Mg:Al = 3 : 0 : 2, (c-d) Cu:Mg:Al = 3 : 0.1 : 2**, (e-f)** Cu:Mg:Al = 3 : 0.2 : 2, **(g-h)** Cu:Mg:Al = 3 : 0.5 : 2, **(i-j)** Cu:Mg:Al = 3 : 0.8 : 2, **(k-l)** Cu:Mg:Al = 3 : 1 : 2. For the precursors co-precipitated with NaOH, all SEM images reveal a closely packed structure regardless of Mg content. Co-precipitation using 2 M NaOH as the precipitation agent leads to rapid nucleation and lower growth of the formed nuclei due to the much higher supersaturation. During calcination the closely packed nanoparticles experience a high degree of sintering due to their proximity and size and formation of large grains.

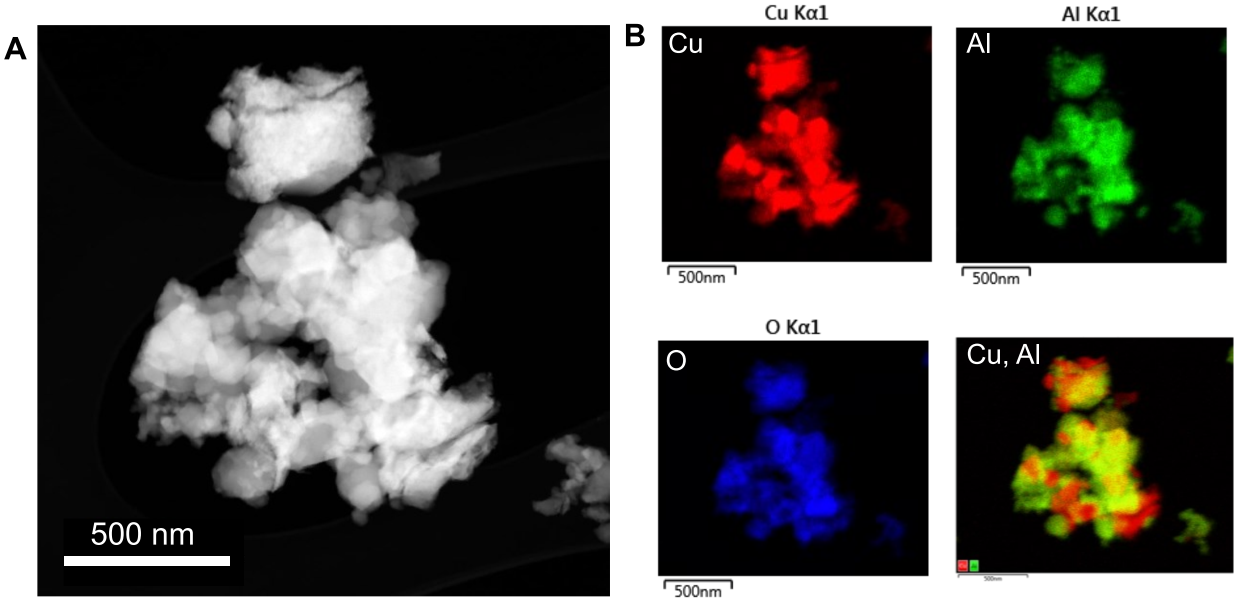

Supplementary Fig. S10. Characterization of CuAl mixed metal oxides derived from CuAl-LDH without residual sodium. (A) HAADF-STEM image of CuAl-MMO calcined at 950°C. (B) EDX mapping showing the distribution of Cu (Red), Al (Green), and O (Blue), and overlay map of Cu and Al. The CuAl-LDH sample was prepared by precipitation with an aqueous solution containing 2M NaOH.

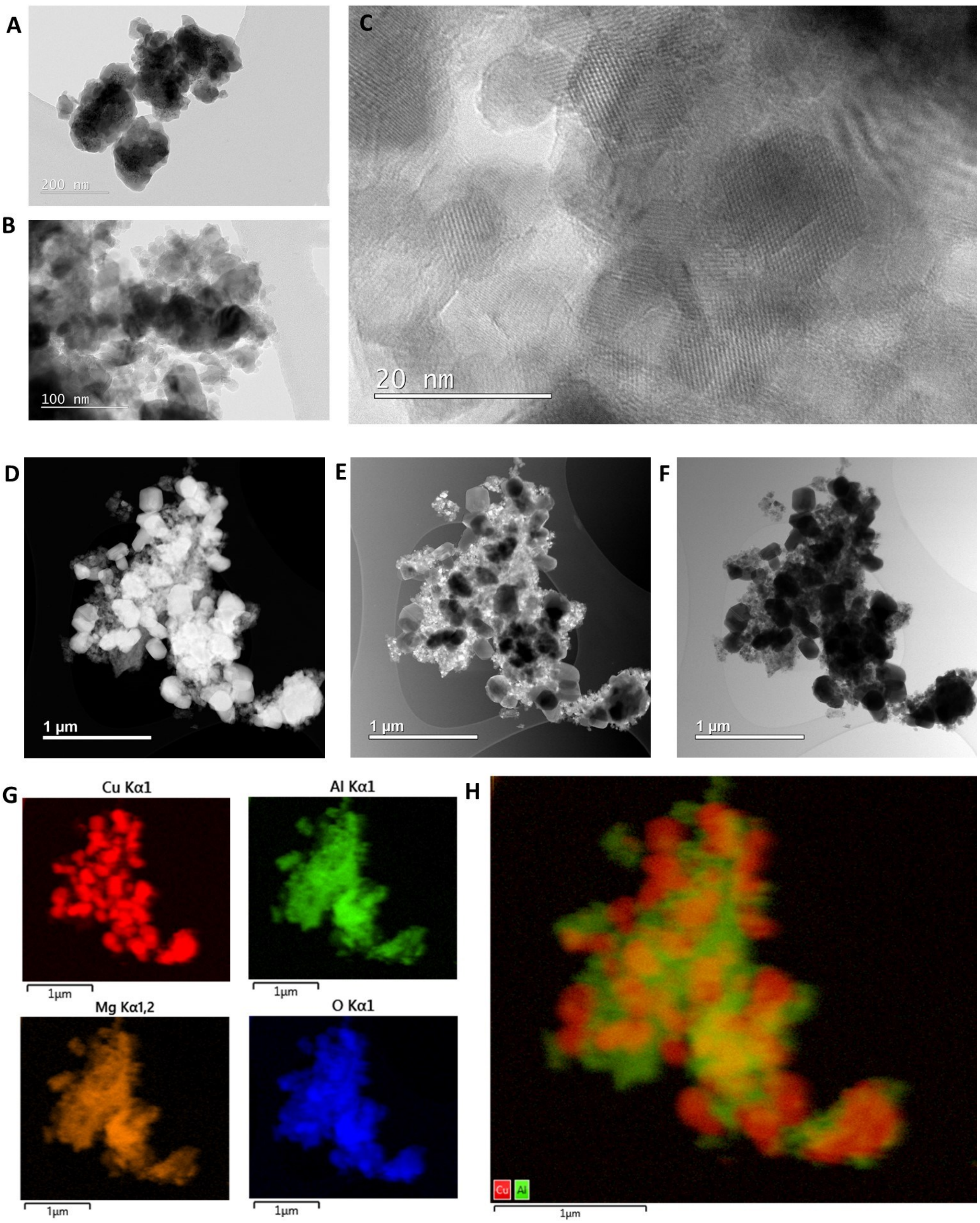

Supplementary Fig. S11. Characterization of CuMgAl-LDH precursor and calcined mixed metal oxides. **(A)** STEM image of CuMgAl-LDH precursor. **(B)** STEM image and **(C)** HRTEM image of CuMgAl mixed metal oxides calcined at 800°C for 3 h (Cu:Mg:Al = 3:1:2). **(D)** HAADF-STEM, **(E)** BF-STEM and **(F)** STEM images of CuMgAl-MMO calcined at 950 °C. **(G)** EDX mapping showing the distribution of Cu (Red), Al (Green), Mg (Yellow), and O (Blue), and **(H)** overlay of Cu and Al. The CuMgAl-LDH sample was prepared by precipitation with an aqueous solution containing 2M NaOH.

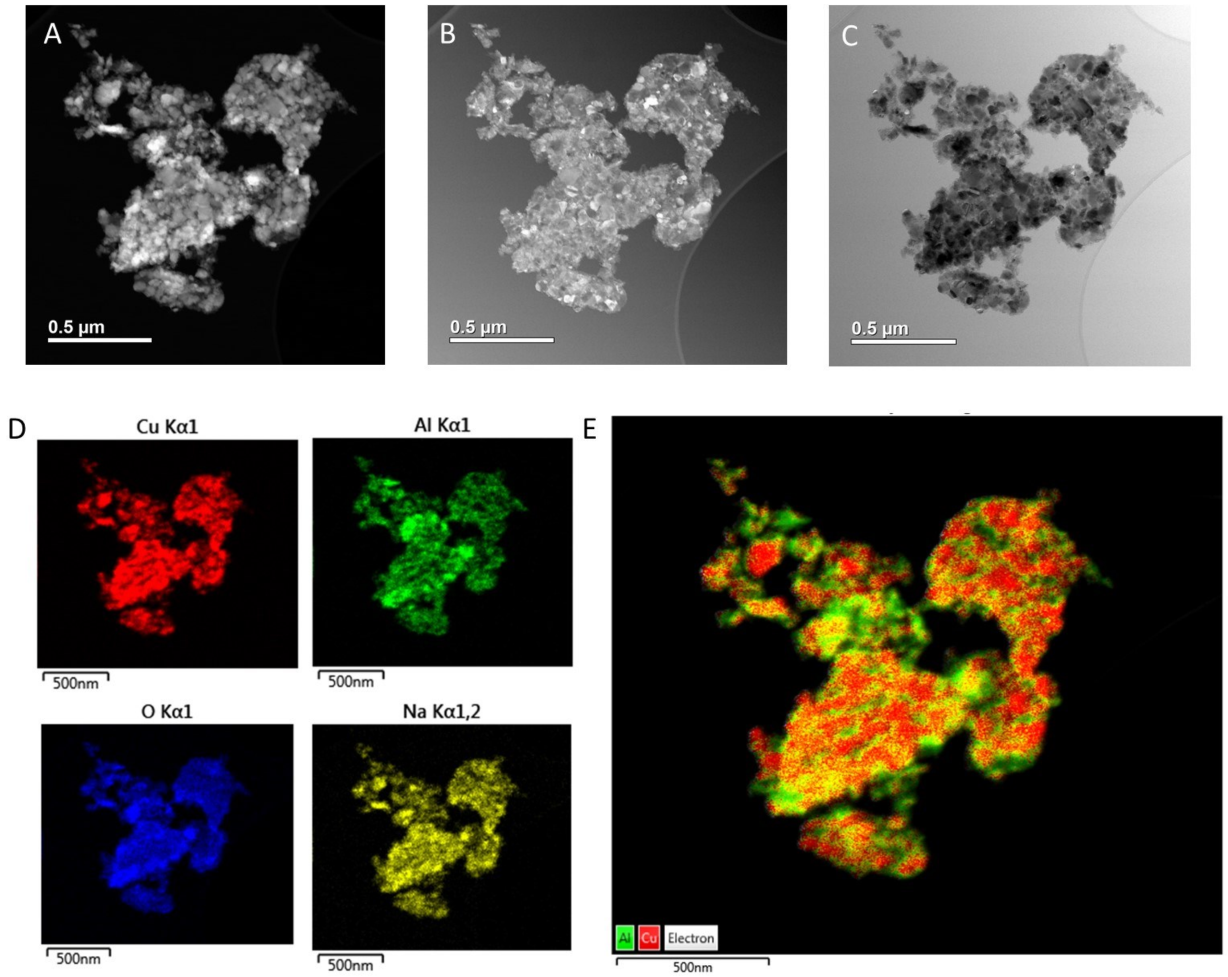

Supplementary Fig. S12. Characterization of sodium-stabilized CuAl mixed metal oxides derived from CuAl-LDH. (A) HAADF-STEM, **(B)** BF-STEM and **(C)** STEM images of CuAl-MMO calcined at 950°C. **(D)** EDX mapping showing the distribution of Cu (Red), Al (Green), Na (Yellow), and O (Blue), and **(E)** overlay map of Cu and Al. The CuAl-LDH sample was prepared by precipitation with an aqueous solution containing 1M NaOH and 1M Na_2CO_3.

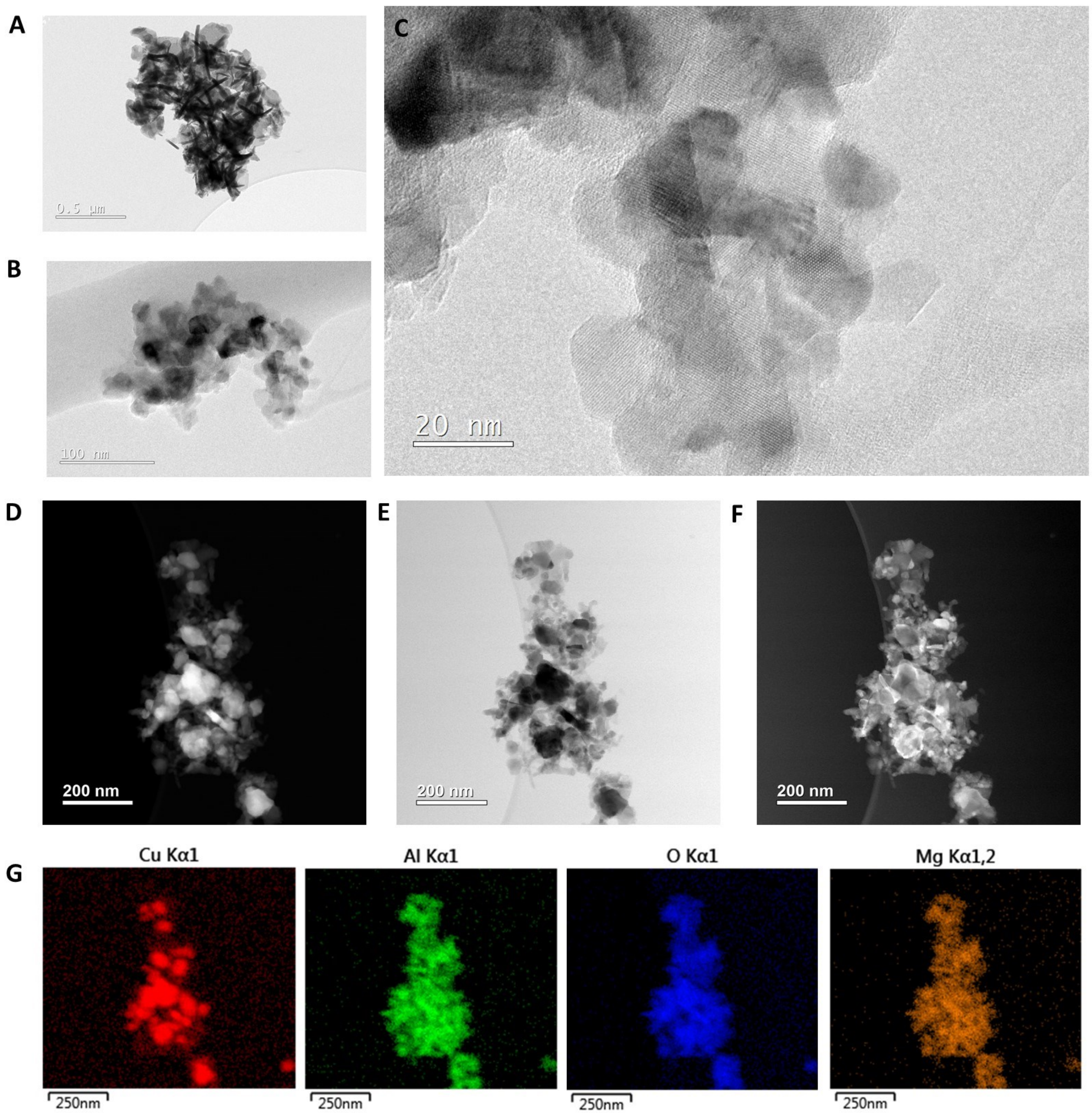

Supplementary Fig. S13. Characterization of Cu-Mg-Al LDH precursor and calcined mixed metal oxides. (A) STEM image of CuMgAl-LDH precursor. (B) STEM image and (C) HRTEM image of CuMgAl mixed metal oxides calcined at 800°C for 3 h (Cu:Mg:Al = 3:1:2). (D) HAADF-STEM, (E) BF-STEM and (F) STEM images of CuMgAl-MMO calcined at 950 °C. (G) EDX mapping showing the distribution of Cu (Red), Al (Green), Mg (Yellow), and O (Blue). The CuMgAl-LDH sample was prepared by precipitation with an aqueous solution containing 1M NaOH and 1M Na_2CO_3.

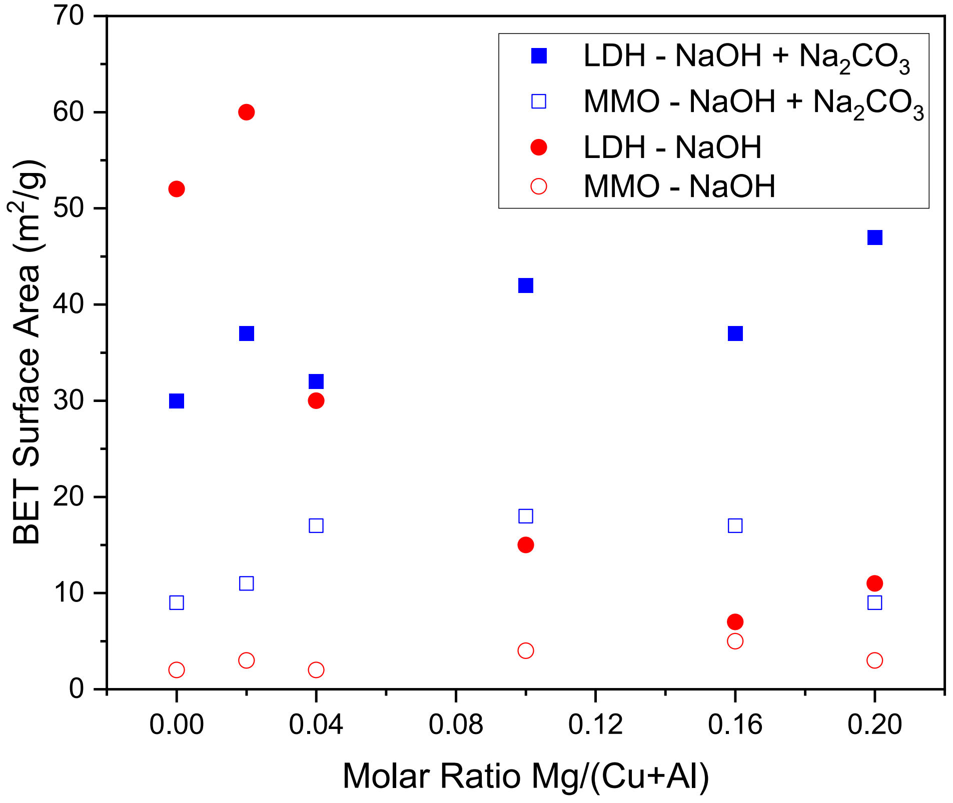

Supplementary Fig. S14. BET surface areas of the precursors and calcined oxygen carriers for varying molar concentrations of Mg. LDH prepared by precipitation using 1M NaOH and 1M Na_2CO_3 are relatively more porous compared to those prepared by precipitation in 2M NaOH.

Supplementary Table S6.

BET surface area measurements for the precursors and calcined samples prepared with NaOH or NaOH + Na_2CO_3 for different [Mg] loading.

Mg/(Cu +Al)	BET surface area (m^2/g) 1M NaOH + 1M Na_2CO_3		BET surface area (m^2/g) 2M NaOH	
	LDH	MMO	LDH	MMO
0	30	9	52	2
0.02	37	11	60	3
0.04	32	17	30	2
0.10	42	18	15	4
0.16	37	17	7	5
0.20	47	9	11	3

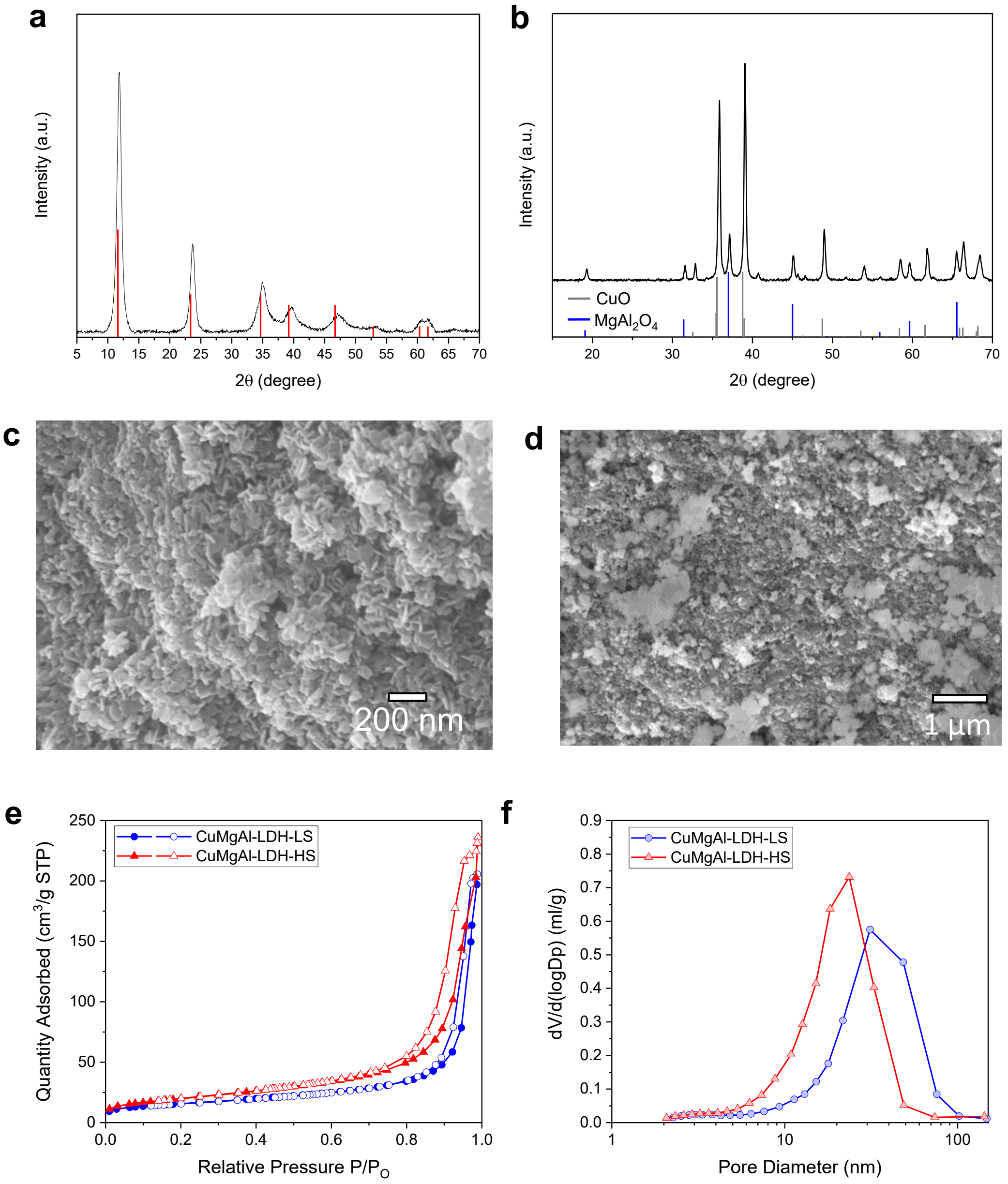

Supplementary Fig. S15. Characterization of CuMgAl LDH precursor and derived mixed oxides. (a) XRD patterns of CuMgAl LDH precursor and (b) derived mixed metal oxides CuMgAl-MMO-HS. Cu:Mg:Al molar ratio is 3:1:2, precipitation at pH of 11 with precipitation agent of 1M NaOH + 1M Na_2CO_3. (c) SEM image of LDH precursor; (d) SEM image of calcined mixed oxides. (e) The N_2 adsorption-desorption isotherms and (f) pore size distributions for the CuMgAl-LDH precursor material. The two isotherms are very similar and follow a Type IV hysteresis loop, characteristic of the N_2 physisorption between aggregates of platelet particles. The pore size distribution narrows and shifts to a lower average pore diameter with an increase in pH value. The initial porosity ($\boldsymbol{\varepsilon_0}$ = 0.74) of the oxygen carrier was calculated by combining He pycnometry and Mercury intrusion porosimetry (MIP). Generally, there is an inverse correlation between the porosity and crushing strength of a particle, therefore to achieve a high crushing strength, a lower porosity was targeted. An increase in synthesis pH from 9.5 to 11 enhanced the crushing strength of calcined mixed metal oxides particles.

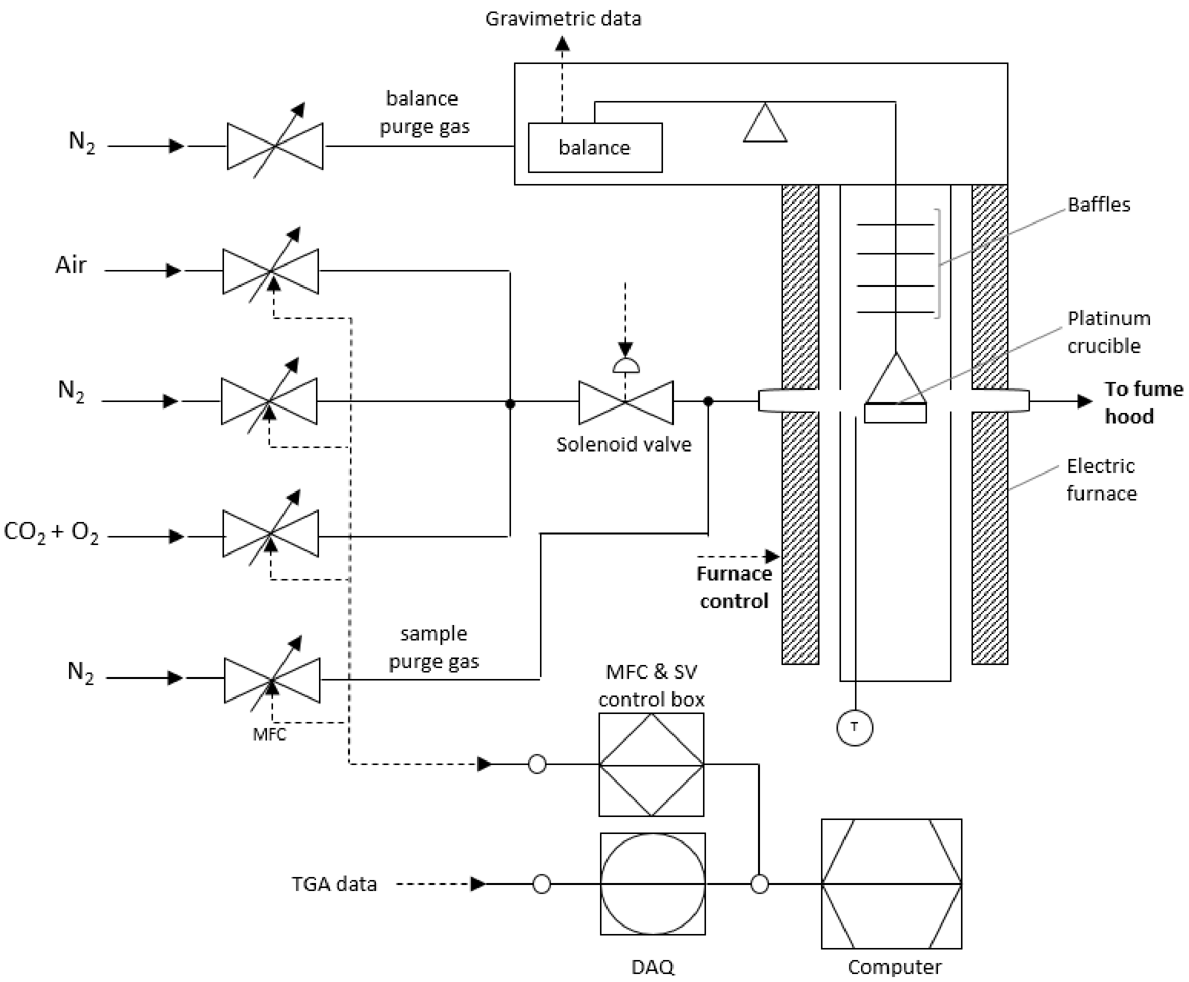

Supplementary Fig. S16. A schematic of the TGA used to study the redox reactions and long-term chemical stability of the oxygen carriers reproduced from Patzschke[30]. Thermogravimetric analysis was carried out using a TA Instruments Q5000 analyser. The TGA software (TA Instruments) controlled the furnace temperature, and sample and balance gas flow rates. The sample weight was measured and recorded by the software every 0.5 s. The atmosphere in the furnace can be controlled by an external programme written in Agilent VEE software, and an external gas supply system consisting of three external mass flow controllers (Bronkhurst).

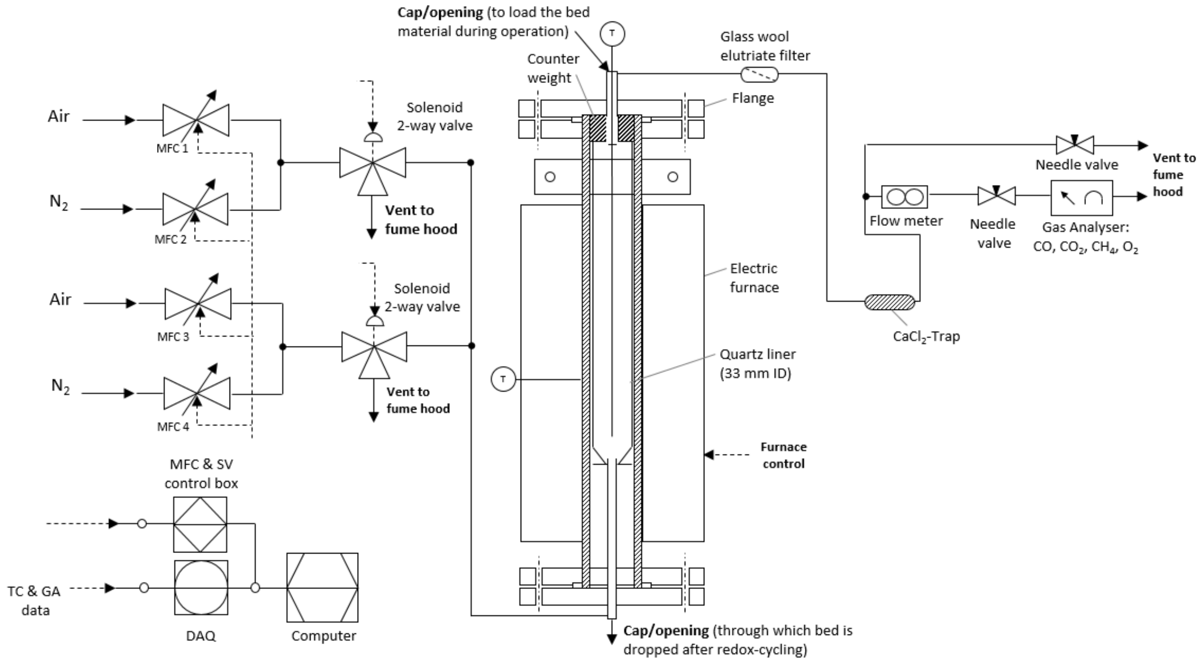

Supplementary Fig. S17. A schematic of the fluidised bed reactor used to study the long-term performance of the oxygen sorbents reproduced from our previous work [30]. A 1.5 kW bench-top fluidised bed reactor (FBR) was used to assess the long-term performance of the oxygen carriers. The fluidised bed reactor is a spouting bed type reactor. The gas supply system is controlled by four mass flow controllers (MFCs) as well as two solenoid valves (used for rapid gas switching). An Agilent VEE code was used to control gas compositions and redox-phase durations for the oxidation and decomposition phases.

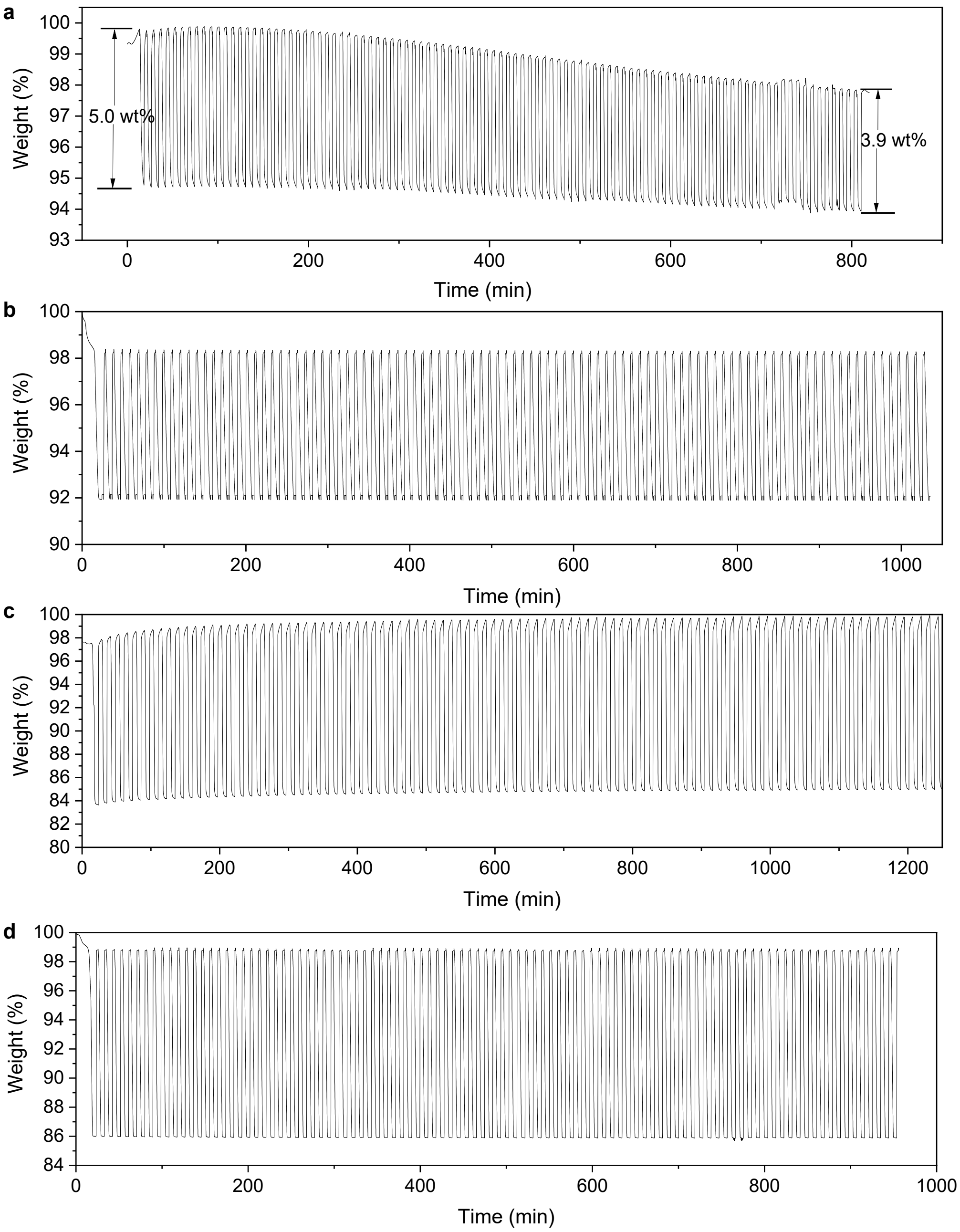

Supplementary Fig. S18. TGA profiles of long-term redox cycling of CuAl-MMO and CuMgAl-MMO. (a-b) Cyclic O_2 release and storage at 900°C for 100 cycles, phase change between CuO and Cu_2O. (a) Cu-Al-MMO, (b) CuMgAl-MMO. The decomposition was carried out under N_2 for 5 min, followed by re-oxidation in air for 3 min. (c-d) Cyclic reduction and oxidation, phase change between CuO and Cu. (c) CuAl-MMO, (d) CuMgAl-MMO. The oxygen sorbents were reduced for 7 min in 5 vol% CO balanced with N_2. This phase was followed by a 1 min N_2 purge. The samples were then re-oxidised for 1 min in air. After a further 1 min in pure N_2, the four-phase sequence was repeated. CuAl-MMO was calcined from CuAl-LDH (molar ratio 3:2) prepared by 2M NaOH. CuMgAl-MMO was calcined from CuMgAl-LDH (molar ratio of 3:1:2) prepared by coprecipitation with 1M NaOH+1M Na_2CO_3.

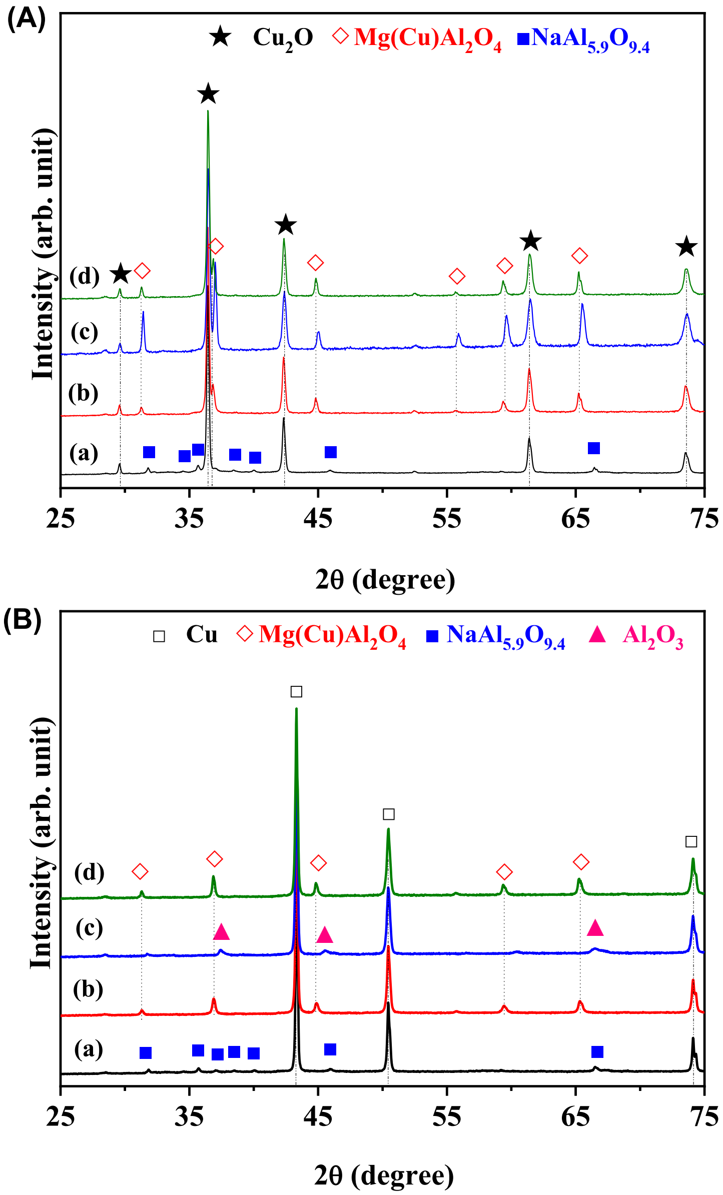

Supplementary Fig. S19. XRD patterns of reduced mixed metal oxides. (A) XRD patterns of mixed metal oxides after O_2 release at 900°C in N_2. **(B)** XRD patterns of reduced MMO at 900°C, in 5 vol. % CO in N_2. **(a)** Cu: Al = 3: 2 (1M NaOH+1M Na_2CO_3); **(b)** Cu: Mg: Al = 3: 1: 2 (1M NaOH+ 1M Na_2CO_3); **(c)** Cu: Al = 3: 2 (2M NaOH); **(d)** Cu: Mg: Al = 3: 1: 2 (2M NaOH). The results show that CuAl-MMO with sodium stabilization (sample a) can be fully reduced to Cu_2O and Cu, respectively, without formation of $CuAl_2O_4$. CuAl-MMO without sodium stabilization (sample c) form a significant amount of $CuAl_2O_4$, which leads to slow kinetics and low oxygen storage capacity. In contrast, CuMgAl-MMO with $MgAl_2O_4$ stabilization (Sample b and d) can be reduced to Cu_2O with minimum amount of $CuAl_2O_4$. The fully reduced sample (CuO to Cu) shows the presence of $MgAl_2O_4$.

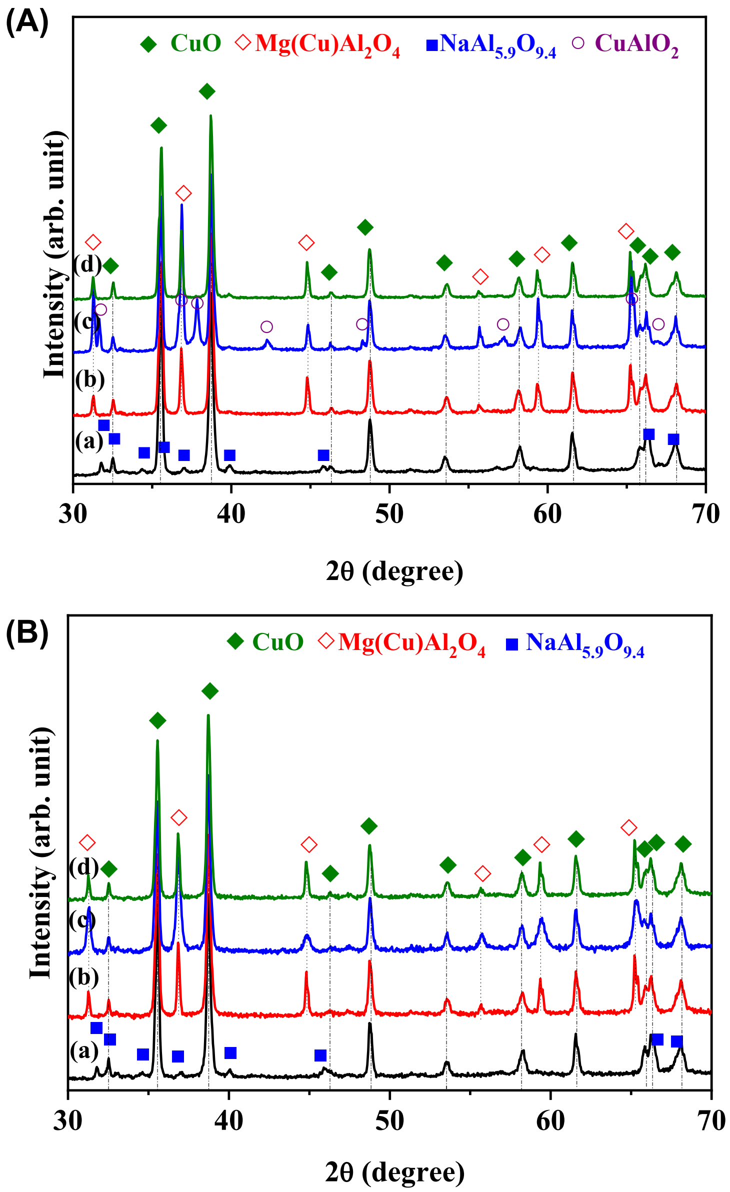

Supplementary Fig. S20. XRD patterns of mixed metal oxides recovered after 100 redox-cycles in the TGA at 900°C. (A) CLOU cycling with decomposition under N_2 and oxidation under air; **(B)** CLC cycling with 5 vol% CO/N_2 for the reduction and oxidation under air. The samples in both plots are: **(a)** Cu : Mg : Al = 3 : 0 : 2 (NaOH + Na_2CO_3), **(b)** Cu : Mg : Al = 3 : 1 : 2 (NaOH + Na_2CO_3), **(c)** Cu : Mg : Al = 3 : 0 : 2 (NaOH), and **(d)** Cu : Mg : Al = 3 : 1 : 2 (NaOH). The XRD plots below indicate the crystalline phases of the oxygen carriers recovered after redox-cycling in the TGA over 100 redox-cycles, on the top at conditions relevant for the CLOU process, on the bottom at CLC conditions. Na-stabilized CuAl-MMO (sample a) did not show formation of $CuAl_2O_4$ or $CuAlO_2$ during redox cycles. CuAl-MMO without sodium promotion (sample c) form a significant amount of $CuAl_2O_4$ and $CuAlO_2$, which leads to slow kinetics and low oxygen storage capacity. CuMgAl-MMO with $MgAl_2O_4$ stabilization (Sample b and d) did not form $CuAlO_2$.

CLOU Reaction Mechanism of CuAl-MMO

The thermodynamics of the Cu-Al-O system was studied to improve the understanding of the oxygen storage and release reactions of the CuAl-MMO. A phase diagram was produced using the Gibbs free energy data reported by Jacob & Alcock[31]. From Supplementary Fig. S21, calcination of the 1:1 Cu:Al LDH precursor in air at 950 °C produces mixed metal oxides with CuO supported on copper aluminate spinel ($CuAl_2O_4$), as confirmed by powder XRD analysis (Fig.5e, fresh material). During redox cycling at 900 °C the oxygen partial pressure is sufficiently low for the active cupric oxide (CuO) to decompose to cuprous oxide (Cu_2O) and oxygen through reaction (13),

$$4CuO \rightleftharpoons 2Cu_2O + O_2 \tag{13}$$

The CuO could also react with $CuAl_2O_4$ to form a delafossite-type material ($CuAlO_2$) and oxygen through reaction (14),

$$2CuAl_2O_4 + 2CuO \rightleftharpoons 4CuAlO_2 + O_2 \tag{14}$$

The $CuAl_2O_4$ could also react to form $CuAlO_2$ and alumina (Al_2O_3) and oxygen through reaction (15),

$$4CuAl_2O_4 \rightleftharpoons 2CuAlO_2 + 2Al_2O_3 + O_2 \tag{15}$$

Although all three reactions are thermodynamically feasible, the absence of $CuAlO_2$ in the XRD pattern of the O_2 released sample (Fig. 5e) and appearance in the cycled material (Fig. 5e) indicates reaction (2) was kinetically limited, as observed in literature[32]. The $CuAlO_2$ peak in the cycled material confirms some of the active CuO was not regenerated during re-oxidation, representing a loss in oxygen storage capacity over 100 cycles as observed in Fig. 5c. The oxidation of $CuAlO_2$ (reverse reaction (14)) is limited by kinetics and not thermodynamics, and therefore the active CuO may be recovered if the segment time was increased, however this would severely impact the operation and economics of commercial chemical looping units. The absence of Al_2O_3 from all XRD patterns (Fig. 5e) indicates reaction (15) did not proceed at an appreciable rate.

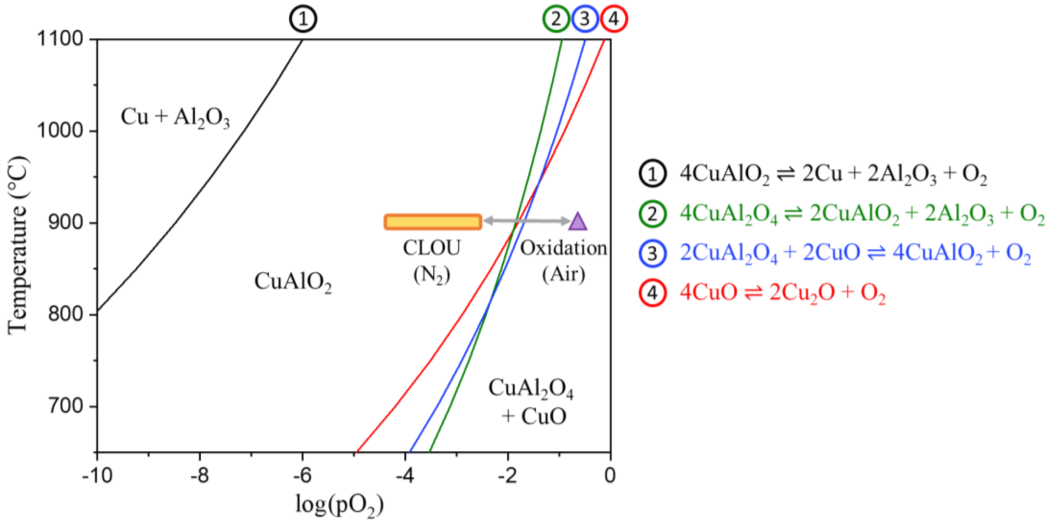

Supplementary Fig. S21. Cu-Al-O system equilibrium phase diagram for a 1:1 molar mixture of CuO and Al_2O_3 based on data reported by Jacob & Alcock[31]. The redox cycling conditions used in this work (Fig. 5 and 6, Supplementary Fig. S18) are demarcated by the CLOU and oxidation regions.

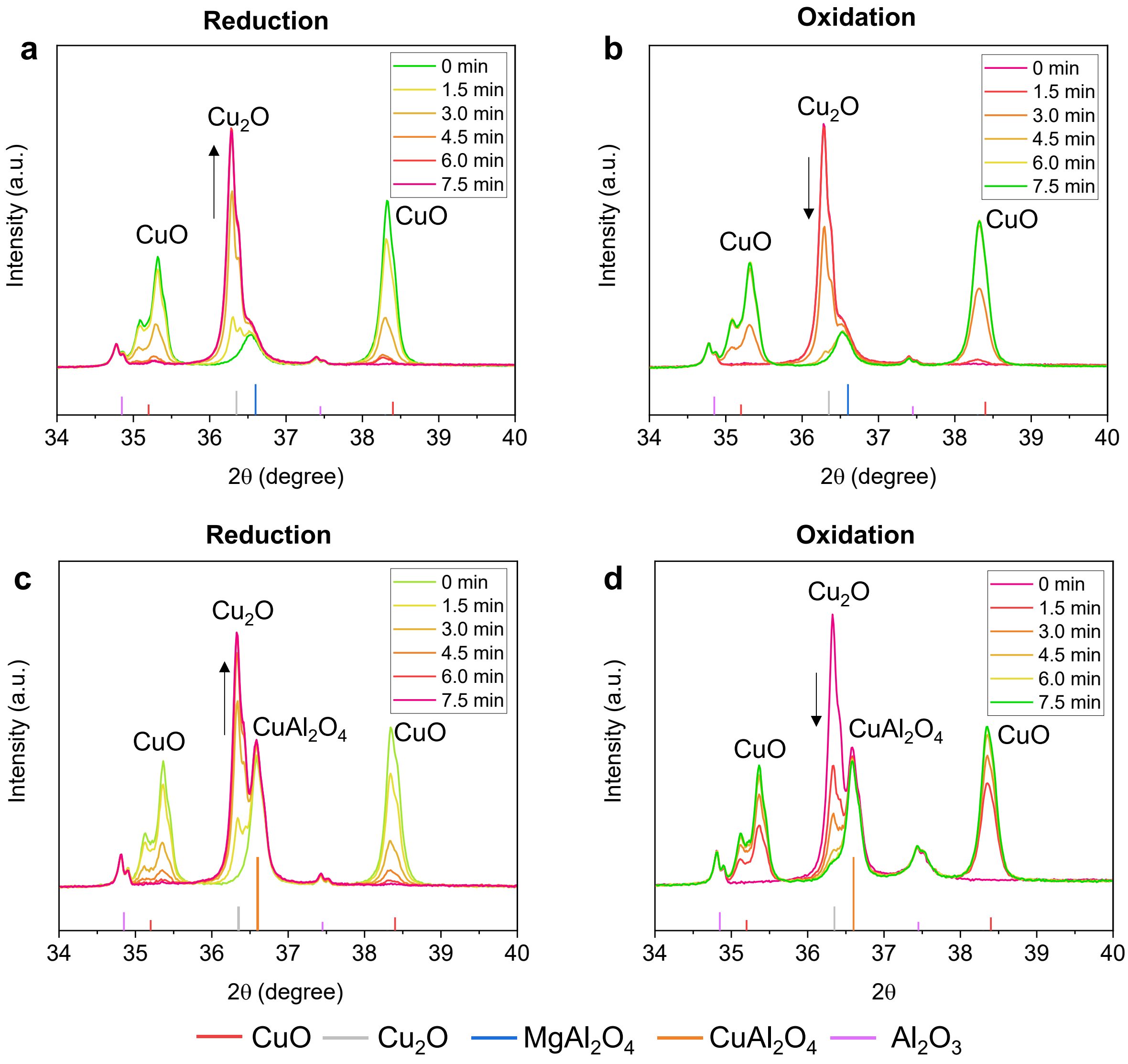

Supplementary Figure S22. ***In situ* high temperature XRD analyses.** (**a-b**) XRD patterns of CuMgAl-MMO with decomposition in N_2 (**a**) and oxidation in air (**b**). Sample derived from CuMgAl-LDH (molar ratio is 3:1:2) with precipitation agent of 1M NaOH + 1M Na_2CO_3. (**c-d**) XRD patterns of CuAl-MMO, with decomposition in N_2 (**c**) and oxidation in air (**d**). As observed from the XRD patterns, the CuMgAl-MMO shows reversible phase changes. CuAl-MMO shows the formation of $CuAl_2O_4$, which will slowly transform to $CuAlO_2$ in redox cycles as shown in Figure 5 in the main paper.

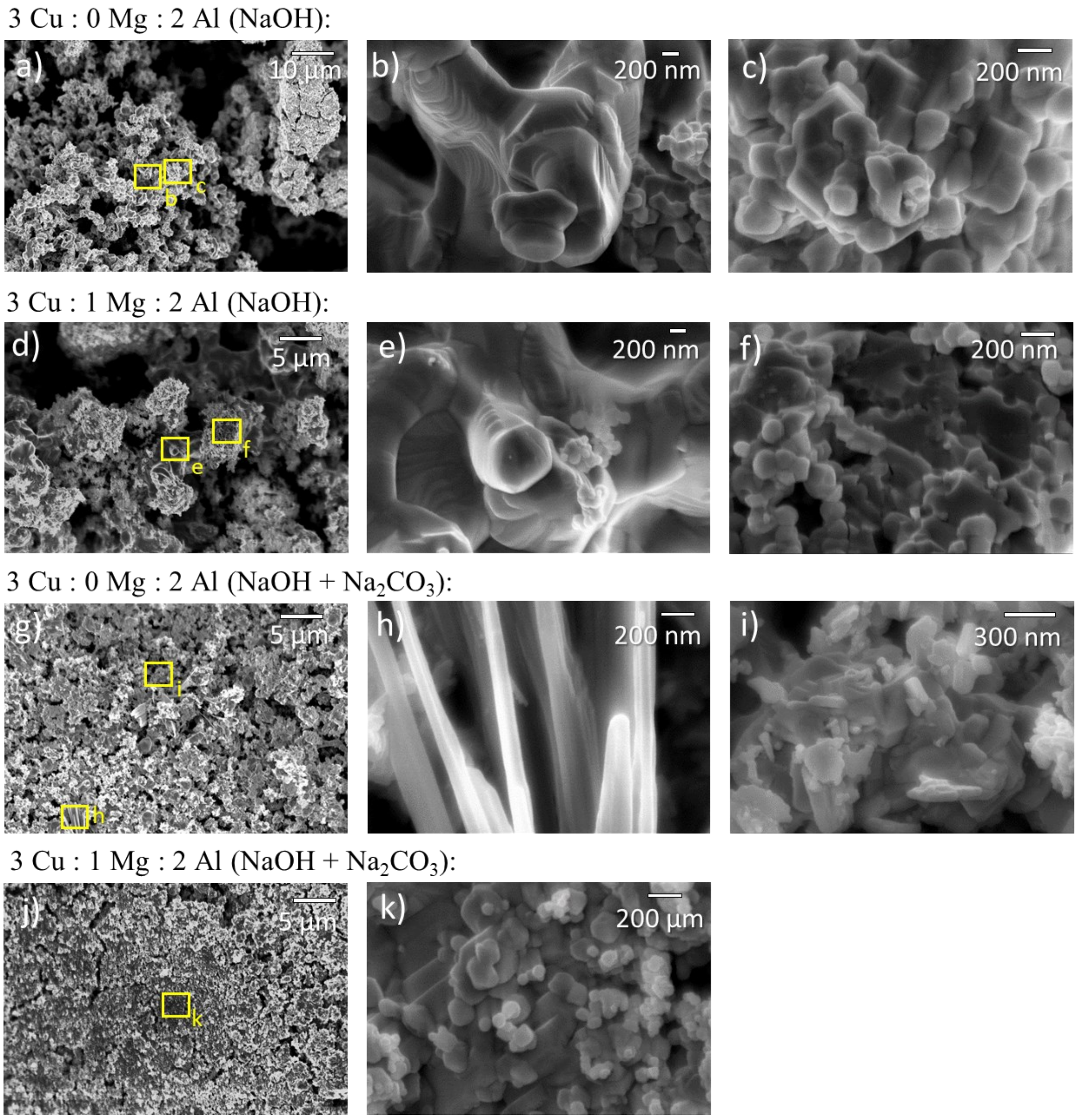

Supplementary Fig. S23. SEM images of samples recovered after 100 CLOU cycles in the TGA at 900 °C using N_2 and air for the decomposition and oxidation, respectively: (a-c) CuAl-MMO (3:2) prepared with 2M NaOH; **(d-f)** CuMgAl-MMO (3:1:2) prepared with 2M NaOH; **(g-i)** CuAl-MMO(3:2) prepared with 1M NaOH + 1M Na_2CO_3; **(j, k)** CuMgAl-MMO (3:1:2) prepared with 1M NaOH + 1M Na_2CO_3; The needle-like features observed in (h) were determined to be rich in Na by SEM-EDS (potentially Na-Al-O oxides). Contrary to the sample shown in **(g-i)**, no needle-like features were found in the sample shown in **(j, k),** which matches with the results from XRF and ICP (that no sodium had contaminated the calcined CuMgAl-MMO sample).

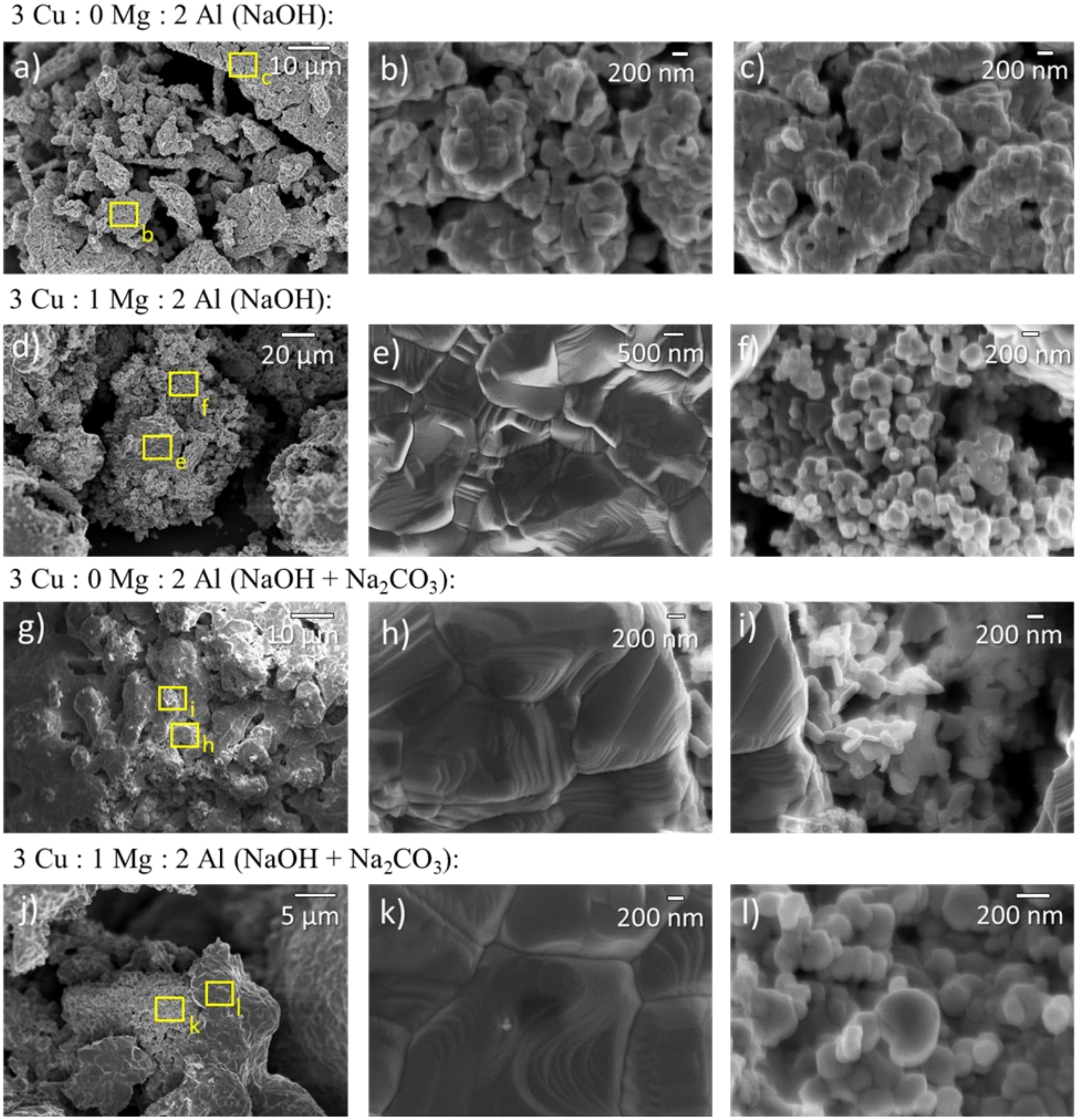

Supplementary Fig. S24. SEM images of samples recovered from TGA experiments with full reduction under CO/N_2 and oxidation in air after 100 redox-cycles at 900 °C: (a-c) CuAl-MMO(3:2) prepared with 2M NaOH; **(d-f)** CuMgAl-MMO (3:1:2) prepared with 2M NaOH; **(g-i)** CuAl-MMO(3:2) prepared with 1M NaOH + 1M Na_2CO_3; **(j-l)** CuMgAl-MMO (3:1:2) prepared with 1M NaOH + 1M Na_2CO_3; In general, large grains were formed due to sintering, while nanoplatelet-like morphology can be observed locally for samples derived from LDH precursors.

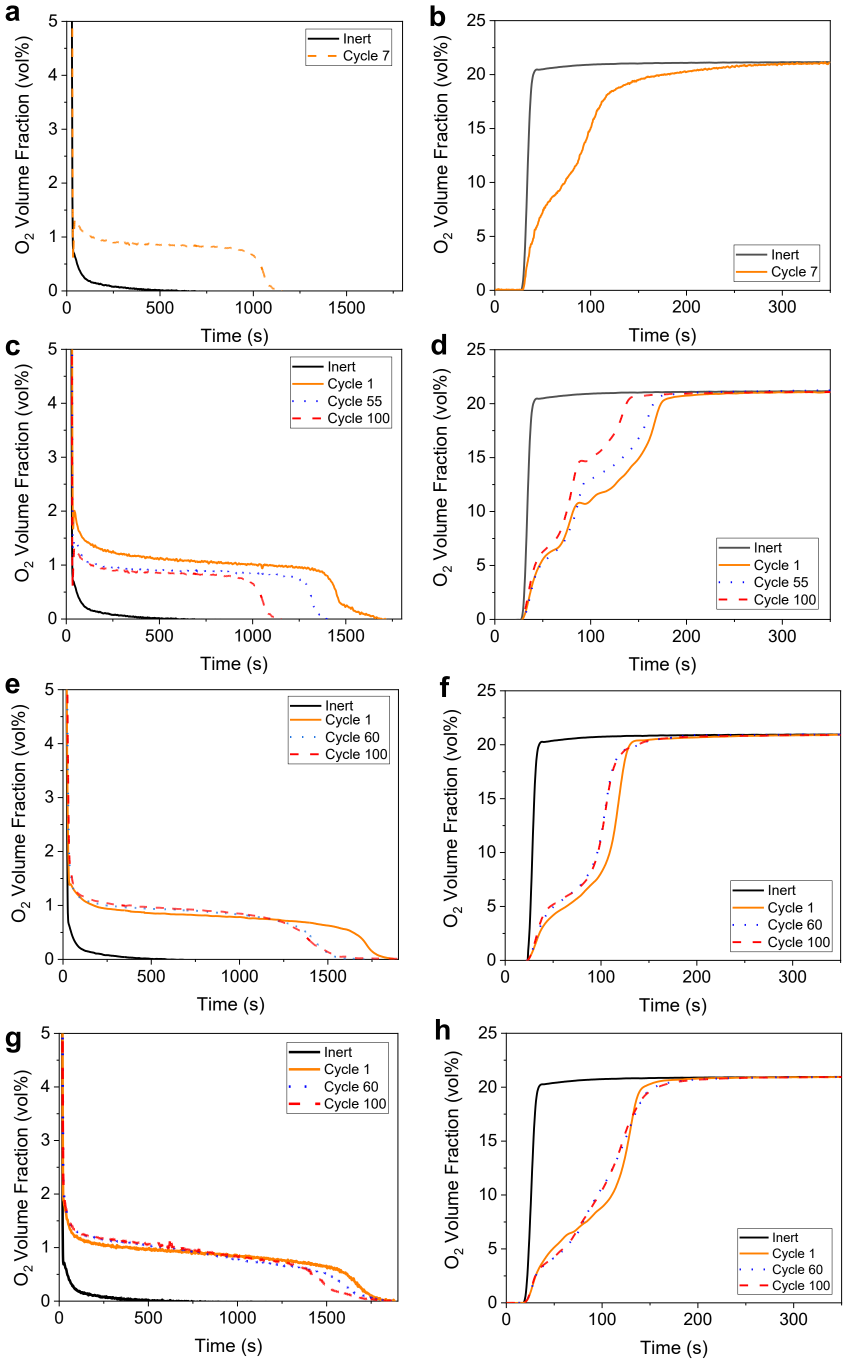

Supplementary Fig. S25. Oxygen profiles for the oxygen release and oxidation reactions at 900°C, (a, b) CuAl-MMO, **(c, d)** CuMgAl-MMO-LS (PC), **(e, f)** CuMgAl-MMO-HS (PC), **(g, h)**, and CuMgAl-MMO-HS (FC), respectively. Where PC is partial cycling (100 cycles of partial decomposition and oxidation with full cycles every 20 cycles), and FC is full cycling (100 cycles of full decomposition and oxidation). $u/u_{mf} = 5$. Here we show the oxygen release and oxidation oxygen concentration profiles for the long-term cycling experiments in the FBR. It can be seen that the CuAl-MMO releases only 70% of the theoretical oxygen, indicating extreme attrition after 10 cycles in the FBR. The CuMgAl-MMO-LS profiles clearly show continual attrition over 100 cycles of partial cycling, whereas the CuMgAl-MMO-HS profiles become stable. A similar behaviour can be seen for the CuMgAl-MMO-HS during full cycling operation in the FBR.

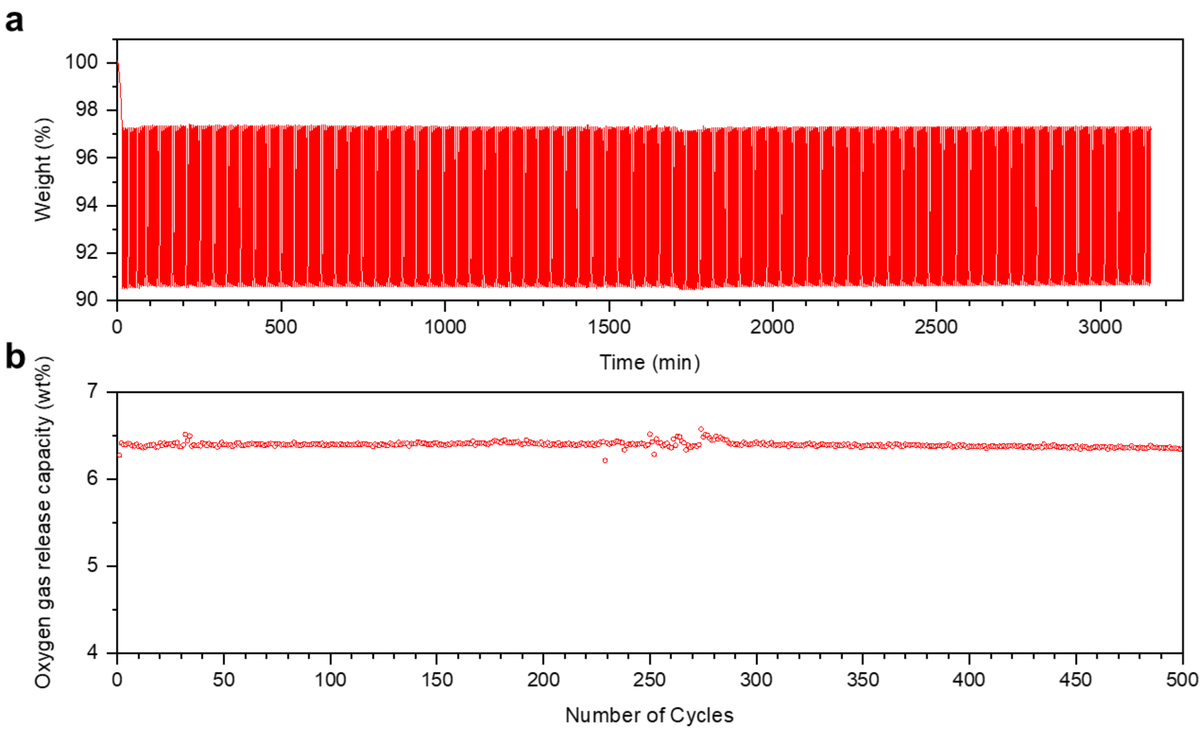

Supplementary Fig. S26. TGA profiles of long-term redox cycling of CuMgAl-MMO-HS. (a) Cyclic O_2 release and storage at 900°C for 500 cycles, phase change between CuO and Cu_2O. (b) O_2 release capacity over 500 cycles of O_2 release.

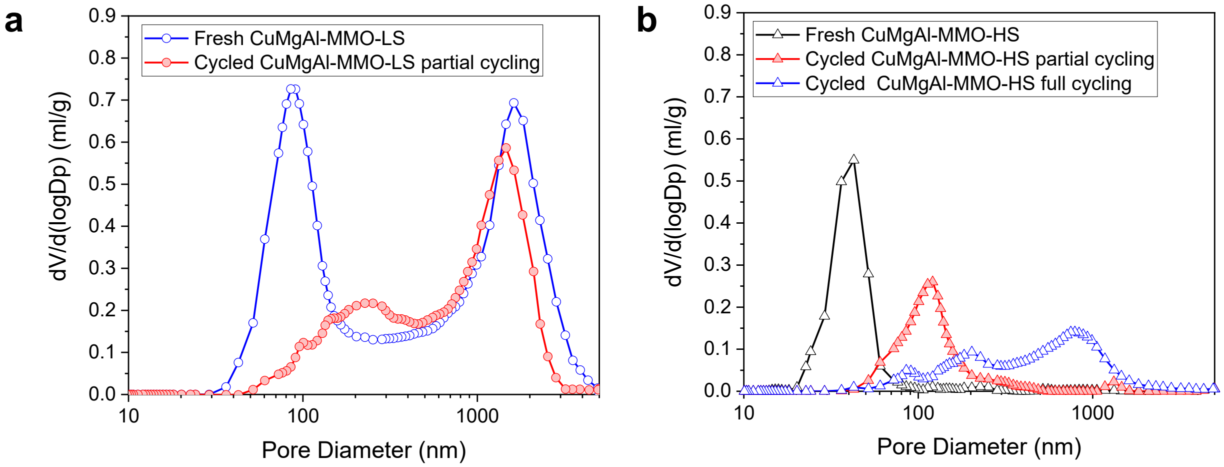

Supplementary Fig. S27. Pore structural analysis of fresh and cycled mixed oxides derived from mercury intrusion pycnometry (MIP). (a) pore size distribution of fresh CuMgAl-MMO-LS and cycled in FBR reactor, **(b)** pore size distribution of fresh CuMgAl-MMO-HS and cycled samples in FBR reactor. Fresh: freshly calcined, partial cycling: 100 cycles of partial decomposition and oxidation with full cycles every 20 cycles, full cyling: 100 cycles of full decomposition and oxidation. The change of pore size distribution derived from MIP indicated the decrease in average pore diameter (towards the mesoporous range) for the mixed metal oxides derived from LDH synthesised at relatively high pH, but also a shift from bi-modal to uni-modal pore volume distribution. The evolution of the pore size distribution indicates that the mechanical properties of the metal oxide materials remain to be improved in order to be used in fluidized bed reactors. While the focus of this study is to demonstrate the materials chemistry and redox properties of LDH-derived mixed metal oxides, cutting-edge particle engineering techniques can be used to enhance the mechanical properties, which will be explored in the future work.

Supplementary Table S7.

Results of Reitveld refinement of XRD patterns of CuMgAl-MMO-HS, freshly calcined, after 100 cycles of partial decomposition and oxidation, and 100 cycles of full decomposition and oxidation.

	composition, wt%	
Sample	CuO	$MgAl_2O_4$
CuMgAl-MMO-HS fresh	60.35 ± 0.05	39.65 ± 0.44
CuMgAl-MMO-HS partial cycling	60.33 ± 0.51	39.67 ± 0.59
CuMgAl-MMO-HS full cycling	60.32 ± 0.05	39.68 ± 1.06

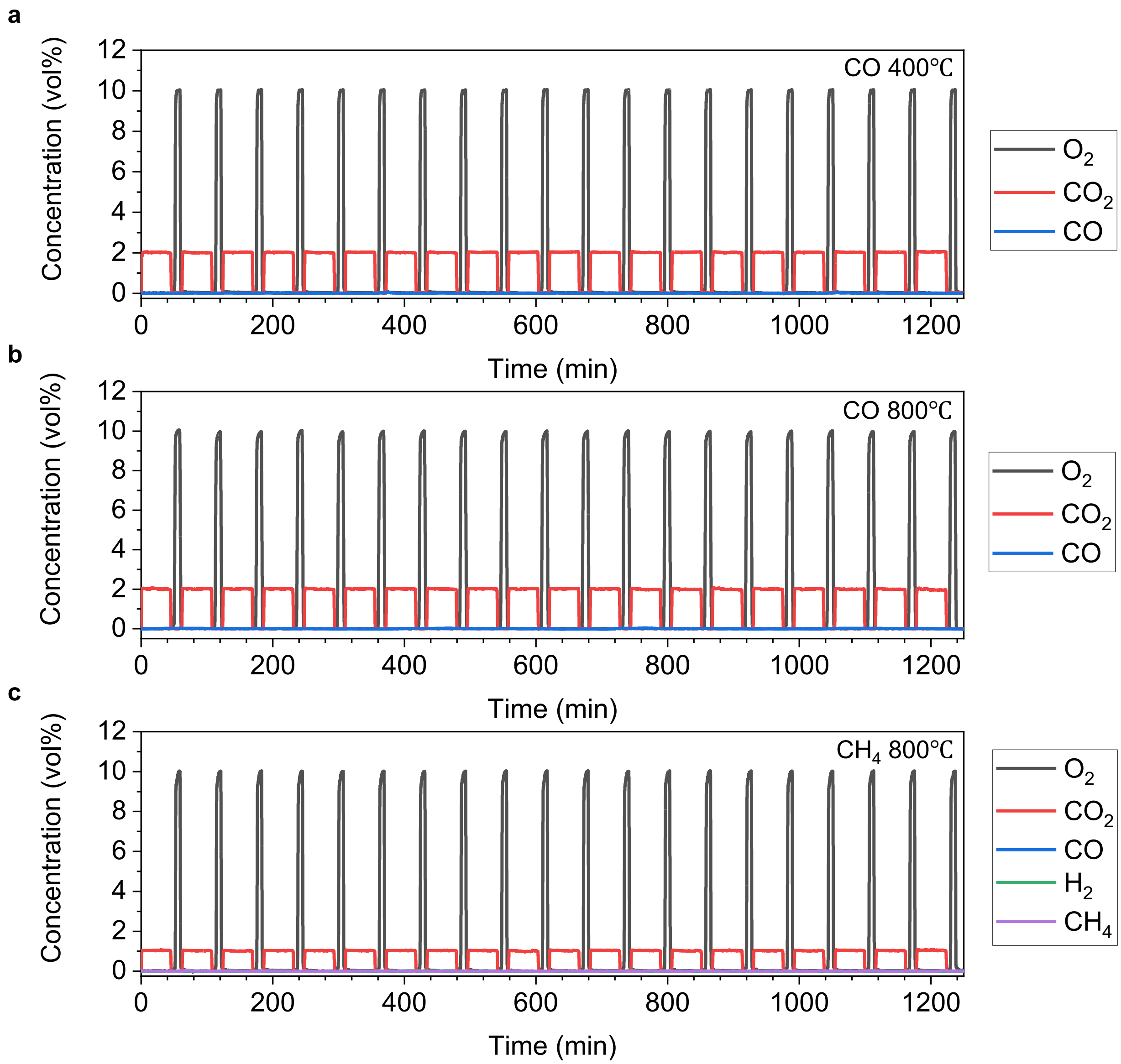

Supplementary Fig. S28. Gas concentration profiles of chemical looping combustive purification (CLCP) cycling of CuMgAl-MMO in FBR. (a) 2 vol% CO/N_2 used for the reduction and oxidation under 10 vol% O_2/N_2 at 400℃, (b) 2 vol% CO/N_2 used for the reduction and oxidation under 10 vol% O_2/N_2 at 800℃, (c) 1 vol% CH_4/N_2 used for the reduction and oxidation under 10 vol% O_2/N_2 at 800℃.

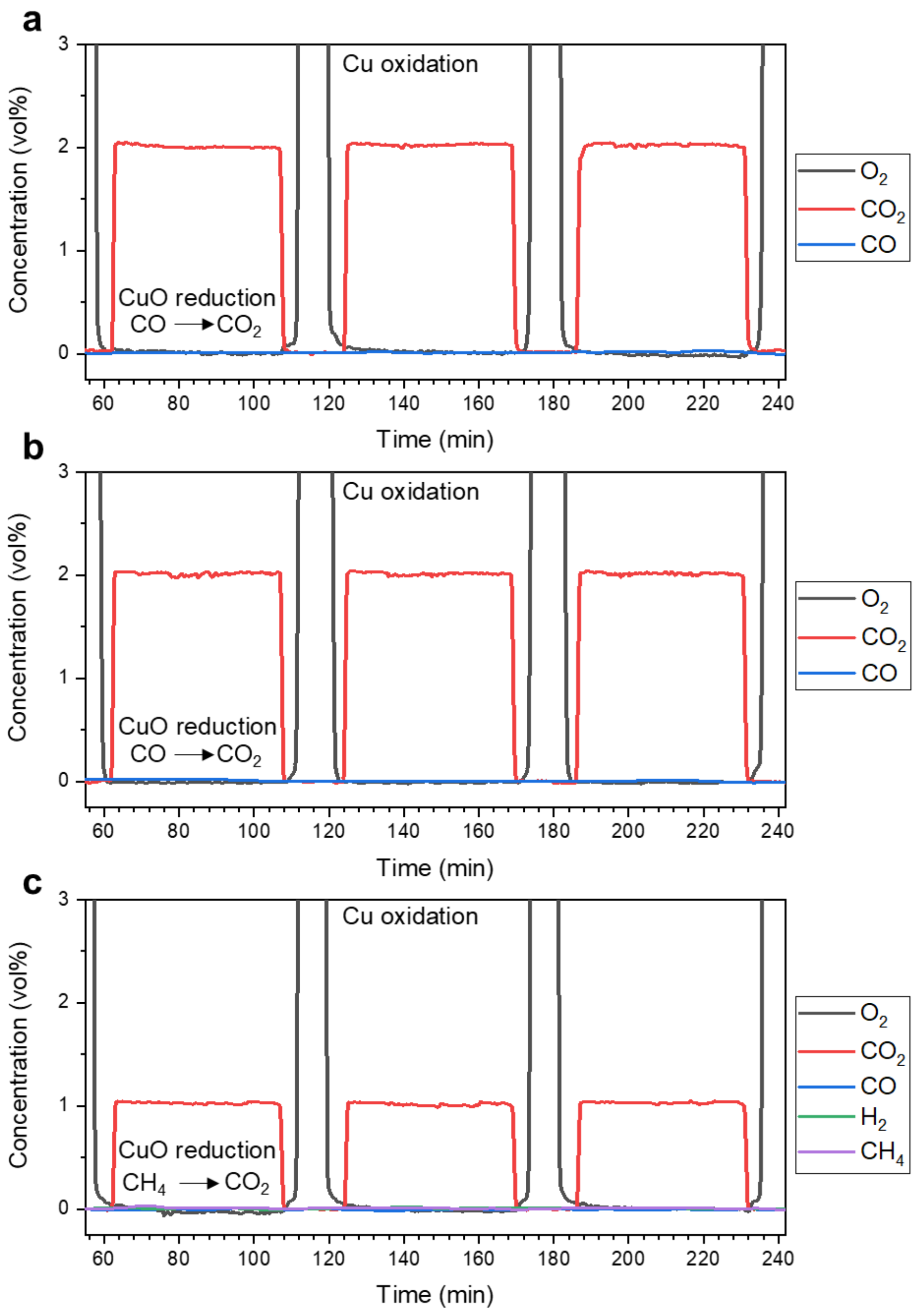

Supplementary Fig. S29. Enhanced gas concentration profiles of chemical looping combustive purification (CLCP) cycling of CuMgAl-MMO in FBR. (a) 2 vol% CO/N_2 used for the reduction and oxidation under 10 vol% O_2/N_2 at 400℃, (b) 2 vol% CO/N_2 used for the reduction and oxidation under 10 vol% O_2/N_2 at 800℃, (c) 1 vol% CH_4/N_2 used for the reduction and oxidation under 10 vol% O_2/N_2 at 800°C. The intrinsic accuracy of the measurement is 1.0% of reading, and the detection limits of the MGA3000C Multi-Gas Analyser (ADC Gas Analysis) are 500 ppm for CO, CO_2 and CH_4 and 1000 ppm for O_2.

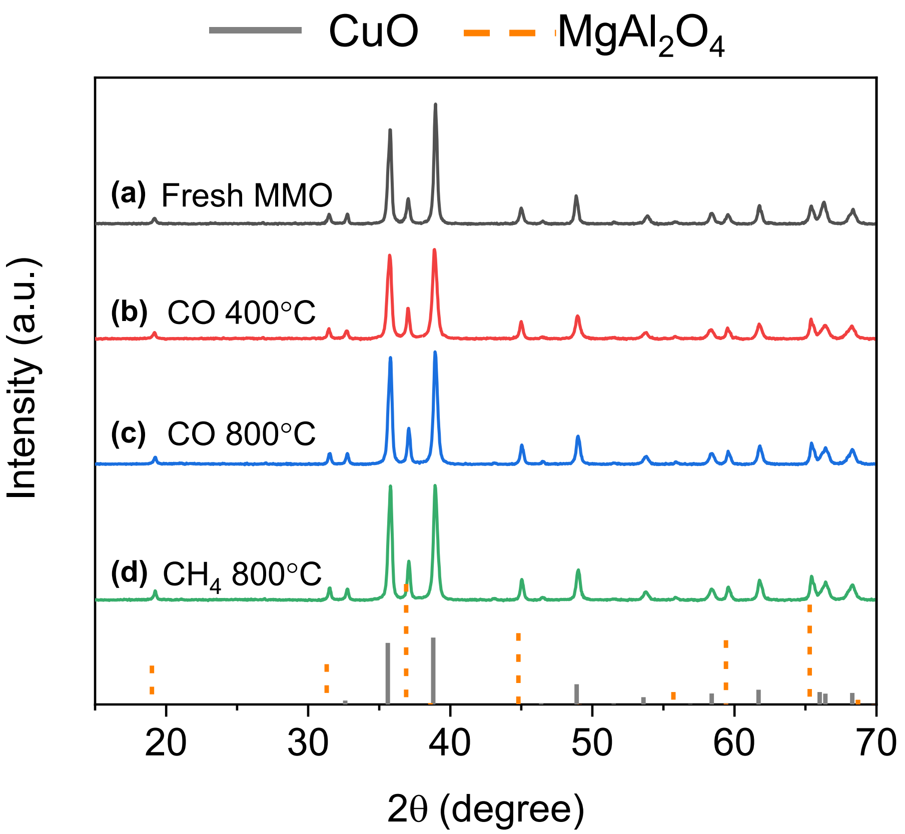

Supplementary Fig. S30. XRD patterns of fresh and cycled CuMgAl mixed metal oxides recovered after 20 redox-cycles in FBR. (a) fresh CuMgAl-MMO-HS, (b) CuMgAl-MMO-HS recovered after 20 CLC cycles in FBR at 400℃ with 2 vol% CO/N_2 for the reduction and oxidation under 10 vol% O_2/N_2, (c) CuMgAl-MMO-HS recovered after 20 CLC cycles in FBR at 800℃ with 2 vol% CO/N_2 for the reduction and oxidation under 10 vol% O_2/N_2, (d) MMO recovered after 20 CLC cycles in FBR at 800℃ with 1 vol% CH_4/N_2 for the reduction and oxidation under 10 vol% O_2/N_2. The XRD plots did not show formation of $CuAl_2O_4$ or $CuAlO_2$ after cycling.

References

1. Song, Q. et al. A high performance oxygen storage material for chemical looping processes with CO_2 capture. *Energy Environ. Sci.* **6**, 288-298 (2013).
2. Mattisson, T., Lyngfelt, A. & Leion, H. Chemical-looping with oxygen uncoupling for combustion of solid fuels. *Int. J. Greenhouse Gas Control* **3**, 11-19 (2009).
3. Arjmand, M., Azad, A.M., Leion, H., Lyngfelt, A. & Mattisson, T. Prospects of Al_2O_3 and $MgAl_2O_4$-supported CuO oxygen carriers in chemical-looping combustion (CLC) and chemical-looping with oxygen uncoupling (CLOU). *Energy Fuels* **25**, 5493-5502 (2011).
4. Gayán, P. et al. Development of Cu-based oxygen carriers for Chemical-Looping with Oxygen Uncoupling (CLOU) process. *Fuel* **96**, 226-238 (2012).
5. Mattisson, T., Leion, H. & Lyngfelt, A. Chemical-looping with oxygen uncoupling using CuO/ZrO_2 with petroleum coke. *Fuel* **88**, 683-690 (2009).
6. Arjmand, M., Keller, M., Leion, H., Mattisson, T. & Lyngfelt, A. Oxygen release and oxidation rates of $MgAl_2O_4$-supported CuO oxygen carrier for chemical-looping combustion with oxygen uncoupling (CLOU). *Energy Fuels* **26**, 6528-6539 (2012).
7. Xu, L., Wang, J., Li, Z. & Cai, N. Experimental Study of Cement-Supported CuO Oxygen Carriers in Chemical Looping with Oxygen Uncoupling (CLOU). *Energy Fuels* **27**, 1522–1530 (2013).
8. Wen, Y.Y., Li, Z.S., Xu, L. & Cai, N.S. Experimental Study of Natural Cu Ore Particles as Oxygen Carriers in Chemical Looping with Oxygen Uncoupling (CLOU). *Energy Fuels* **26**, 3919-3927 (2012).
9. Leion, H. et al. Use of $CaMn_{0.875}Ti_{0.125}O_3$ as Oxygen Carrier in Chemical-Looping with Oxygen Uncoupling. *Energy Fuels* **23**, 5276-5283 (2009).
10. Arjmand, M. et al. $Ca_xLa_{1-x}Mn_{1-y}M_yO_{3-\delta}$ (M = Mg, Ti, Fe, or Cu) as oxygen carriers for chemical-looping with oxygen uncoupling (CLOU). *Energy Fuels* **27**, 4097-4107 (2013).
11. Lau, C.Y., Dunstan, M.T., Hu, W., Grey, C.P. & Scott, S.A. Large scale in silico screening of materials for carbon capture through chemical looping. *Energy Environ. Sci.* **10**, 818-831 (2017).
12. Miura, N., Ikeda, H. & Tsuchida, A. $Sr_{1-x}Ca_xFeO_{3-\delta}$ as a New Oxygen Sorbent for the High-Temperature Pressure-Swing Adsorption Process. *Ind. Eng. Chem. Res.* **55**, 3091-3096 (2016).
13. Krzystowczyk, E., Wang, X., Dou, J., Haribal, V. & Li, F. Substituted $SrFeO_3$ as robust oxygen sorbents for thermochemical air separation: correlating redox performance with compositional and structural properties. *Physical Chemistry Chemical Physics* **22**, 8924-8932 (2020).
14. Patzschke, C.F., Boot-Handford, M.E., Song, Q. & Fennell, P.S. Co-precipitated Cu-Mn mixed metal oxides as oxygen carriers for chemical looping processes. *Chem. Eng. J.* **407**, 127093 (2021).
15. Shulman, A., Cleverstam, E., Mattisson, T. & Lyngfelt, A. Chemical – Looping with oxygen uncoupling using Mn/Mg-based oxygen carriers – Oxygen release and reactivity with methane. *Fuel* **90**, 941-950 (2011).
16. Puigdomenech, I. (Royal Institute of Technology, Stockholm, Sweden; 2015).
17. Ingri, N., Kakolowicz, W., Sillén, L.G. & Warnqvist, B. High-speed computers as a supplement to graphical methods—V1Haltafall, a general program for calculating the composition of equilibrium mixtures. *Talanta* **14**, 1261-1286 (1967).
18. Eriksson, G. An algorithm for the computation of aqueous multi-component, multiphase equilibria. *Analytica Chimica Acta* **112**, 375-383 (1979).

19. Johnson, J.W., Oelkers, E.H. & Helgeson, H.C. SUPCRT92: A software package for calculating the standard molal thermodynamic properties of minerals, gases, aqueous species, and reactions from 1 to 5000 bar and 0 to 1000°C. *Computers & Geosciences* **18**, 899-947 (1992).
20. Krupka, K.M., Cantrell, K.J. & McGrail, B.P. (U.S. Department of Energy, 2010).
21. Allison, J.D., Brown, D.S. & Novo-Gradac, K.J. (Environmental Research Laboratory, Office of Research and Development, U.S. Environmental Protection Agency, Athens, Georgia, USA; 1991).
22. Bernard, E., Zucha, W.J., Lothenbach, B. & Mäder, U. Stability of hydrotalcite (Mg-Al layered double hydroxide) in presence of different anions. *Cement and Concrete Research* **152** (2022).
23. Lothenbach, B. & Winnefeld, F. Thermodynamic modelling of the hydration of Portland cement. *Cement and Concrete Research* **36**, 209-226 (2006).
24. Lothenbach, B. et al. Cemdata18: A chemical thermodynamic database for hydrated Portland cements and alkali-activated materials. *Cement and Concrete Research* **115**, 472-506 (2019).
25. Myers, R.J., Lothenbach, B., Bernal, S.A. & Provis, J.L. Thermodynamic modelling of alkali-activated slag cements. *Applied Geochemistry* **61**, 233-247 (2015).
26. Blanc, P., Bourbon, X., Lassin, A. & Gaucher, E.C. Chemical model for cement-based materials: Thermodynamic data assessment for phases other than C–S–H. *Cement and Concrete Research* **40**, 1360-1374 (2010).
27. Johnson, C.A. & Glasser, F.P. Hydrotalcite-like minerals ($M_2Al(OH)_6(CO_3)_{0.5}.xH_2O$, where M = Mg, Zn, Co, Ni) in the environment: synthesis, characterization and thermodynamic stability. *Clays and Clay Minerals* **51**, 1-8 (2003).
28. Takaya, Y., Wu, M. & Kato, Y. Unique Environmental Conditions Required for Dawsonite Formation: Implications from Dawsonite Synthesis Experiments under Alkaline Conditions. *ACS Earth and Space Chemistry* **3**, 285-294 (2019).
29. C. W. Bale, E.B., P. Chartrand, S. A. Decterov, G. Eriksson, A.E. Gheribi, K. Hack, I. H. Jung, Y. B. Kang, J. Melançon, A. D. Pelton, S. Petersen, C. Robelin. J. Sangster and M-A. Van Ende, FactSage Thermochemical Software and Databases (FactSage 7.0). *Calphad* **54**, 35-55 (2016).
30. Patzschke, C.F. in Department of Chemical Engineering, Vol. PhD (Imperial College London, London; 2019).
31. Jacob, K.T. & Alcock, C.B. Thermodynamics of $CuAlO_2$ and $CuAl_2O_4$ and phase equilibria in the system Cu_2O-CuO-Al_2O_3. *Journal of the American Ceramic Society* **58**, 192-195 (1975).
32. Hu, W., Donat, F., Scott, S.A. & Dennis, J.S. The interaction between CuO and Al_2O_3 and the reactivity of copper aluminates below 1000 °C and their implication on the use of the Cu–Al–O system for oxygen storage and production. *RSC Advances* **6**, 113016-113024 (2016).
